# The histone deacetylase SIRT6 blocks myostatin expression and development of muscle atrophy

**DOI:** 10.1038/s41598-017-10838-5

**Published:** 2017-09-19

**Authors:** Sadhana A. Samant, Abhinav Kanwal, Vinodkumar B. Pillai, Riyue Bao, Mahesh P. Gupta

**Affiliations:** 1 Department of Surgery, The Pritzker School of Medicine, The University of Chicago, Chicago, IL 60637 USA; 2 Center for Research Informatics, Biological Sciences Division, The University of Chicago, Chicago, IL 60637 USA

## Abstract

Muscle wasting, also known as cachexia, is associated with many chronic diseases, which worsens prognosis of primary illness leading to enhanced mortality. Molecular basis of this metabolic syndrome is not yet completely understood. SIRT6 is a chromatin-bound member of the sirtuin family, implicated in regulating many cellular processes, ranging from metabolism, DNA repair to aging. SIRT6 knockout (SIRT6-KO) mice display loss of muscle, fat and bone density, typical characteristics of cachexia. Here we report that SIRT6 depletion in cardiac as well as skeletal muscle cells promotes myostatin (Mstn) expression. We also observed upregulation of other factors implicated in muscle atrophy, such as angiotensin-II, activin and Acvr2b, in SIRT6 depleted cells. SIRT6-KO mice showed degenerated skeletal muscle phenotype with significant fibrosis, an effect consistent with increased levels of Mstn. Additionally, we observed that in an *in vivo* model of cancer cachexia, Mstn expression coupled with downregulation of SIRT6. Furthermore, SIRT6 overexpression downregulated the cytokine (TNFα-IFNγ)-induced Mstn expression in C2C12 cells, and promoted myogenesis. From the ChIP assay, we found that SIRT6 controls Mstn expression by attenuating NF-κB binding to the Mstn promoter. Together, these data suggest a novel role for SIRT6 in maintaining muscle mass by controlling expression of atrophic factors like Mstn and activin.

## Introduction

Cachexia, a complex metabolic syndrome, is associated with many end-stage diseases, including congestive heart failure (CHF), chronic kidney disease (CKD), chronic obstructive pulmonary disease (COPD), cancer and AIDS^1^. Cachexia is characterized by severe involuntary loss of body weight that cannot be recovered by exercise and/or nutritional support. Muscle wasting is one of the major consequences of cachexia affecting quality of life leading to morbidity and mortality. Clinically cachectic condition is not only detected as a mere continuous loss of muscle mass and strength, but it is manifested in combination with fatigue, depression, anemia and/or inflammation worsening the prognosis of the underlying disease^2^. Cachexia is reported to be prevalent in 5–15% of CHF and COPD patients, while it rises to 80% in advanced stage of cancer with ~30% cancer patients succumbing to death due to cachexia, rather than the primary disease itself ^3^. In spite of the considerable strides made in the last decade to identify new drug-able targets, no approved therapy is available, so far, for treatment of this debilitating syndrome of cachexia.

An imbalance between the anabolic process of protein synthesis and the catabolic activity of protein degradation is thought to be the primary cause of muscle loss associated with cachexia, or the aging-related sarcopenia. A potent regulator of skeletal muscle mass is myostatin (Mstn), a member of the transforming growth factor (TGF)-β family. Mstn, also known as a growth-differentiation factor 8 (GDF8), is an autocrine/paracrine cytokine, which negatively regulates skeletal muscle mass and growth. Though primarily expressed in skeletal muscle, low level expression of Mstn is also detected in the heart and adipose tissue^4–6^. Mstn is considered as the major muscle atrophy biomarker, because it is directly linked to catabolic signaling associated with muscle wasting, and it is found to be secreted in plasma^6^. Apart from its involvement in skeletal muscle growth, Mstn is also linked to metabolic and cardio-vascular pathologies such as obesity^7^, insulin-resistance^8^, heart failure^9^ and cardiac cachexia^10^. In muscle atrophy originating from cachexia, Mstn levels are elevated. Increased serum levels of Mstn are also observed in large population of patients with chronic heart failure. These patients develop cardiac cachexia (cardiac atrophy) together with skeletal muscle wasting. Mstn is significantly up-regulated in cachectic conditions associated with chronic diseases. In such cachectic state, there is a loss of weight exceeding 6% of edema-free body weight over a period of 6 months, which is also accompanied with metabolic changes^11^. Mstn, being an extra-cellular myokine, mediates its myogenic effects by binding to activin type 2 receptors (Acvr2), which are trans-membrane threonine/serine kinases^12^. Currently, Mstn and Acvr2b are the most studied targets under clinical investigation for developing intervention of cachexia.

At the transcriptional level, several pathways regulate Mstn expression. Mstn promoter is replete with binding motifs for various transcription factors, which include FoxOs, SMADs, and NF-*κ*B as well as with many hormone receptor-binding sites. Accordingly, these factors drive Mstn gene expression either independently or cooperatively^13,14^. However, all the molecular components of the complex signaling pathways for Mstn regulation are not yet completely understood. A recent study has reported that increased expression of Mstn in skeletal muscle from mice with chronic kidney disease could be blocked by subcutaneous injection of an anti-myostatin peptibody, suggesting auto-regulation of Mstn expression^15^. Therefore, given the crucial role of Mstn in regulating muscle mass and its significant contribution to cachexia, blocking its activity in diseases accompanied with muscle loss makes it the most sought out therapeutic target, and holds a lot of promise for clinical application.

The NAD^+^-dependent lysine deacylases, called sirtuins, are implicated in regulation of wide variety of biological functions ranging from cellular growth, stress-resistance, metabolism, genome stability to aging^16^. There are seven evolutionarily conserved mammalian sirtuins (SIRT1-7) distributed to different compartments of the cell. They possess different deacylation activities to post-translationally modulate functions of their targets influencing major cellular pathways^17^. Of the seven, SIRT6 is associated with chromatin and possesses histone deacetylase as well as mono-ADP-ribosylase activities, for both of which it needs NAD^+^ as a co-substrate. SIRT6-deficient (SIRT6-KO) mice display acute degenerative, aging-like (progeroid) phenotype and succumb to death by the age of one month^18^. SIRT6-KO mice exhibit reduced body size and weight with severe hypoglycemia, lymphopenia, loss of bone mineral density (BMD), spinal curvature and loss of subcutaneous fat. Mice depleted of SIRT6 phenotypically resemble a condition akin to cachexia with extensive metabolic problems, inflammation and increased energy expenditure. Chronic inflammation is also considered as one of the underlying causes of pathogenesis of cachexia. The pro-inflammatory cytokines such as tumor necrosis factor-α (TNF-α), interleukin (IL)-1 and 6 are shown to be the primary contributors of inflammatory catabolism during cachexia^19^. TNF-α treatment of muscle cells was found to increase Mstn expression. Such Mstn upregulation as well as TNF-α-mediated degradation of skeletal muscle proteins was shown to be dependent on activation of NF-*κ*B, the pro-inflammatory transcription factor^15,20,21^. NF-*κ*B is also a known inhibitor of myogenesis and muscle regeneration. NF-*κ*B is regulated transiently under physiological conditions; however, it is constitutively activated in skeletal muscles-associated pathologies, such as atrophy/cachexia, muscular dystrophies, rhabdomyosarcomas, inflammatory idiopathic myopathies etc^22^. It has been shown that NF-*κ*B and TNF-α are essential components of the feed forward mechanism for Mstn induction^21^. NF-*κ*B activation is also reported to transcriptionally upregulate *Mstn* expression during cachexia, associated with hepatic cirrhosis^23^. Under physiological conditions, SIRT6 acts as a repressor of NF-*κ*B signaling^24^. In SIRT6-KO mice, over-activation of NF-*κ*B signaling and excessive inflammation was taken as a likely perpetrator for erosive colitis seen in these mice^24^. SIRT6 is considered a longevity factor, and it has been shown to extend healthy life span by curtailing aging associated inflammation by blocking NF-*κ*B signaling.

In this study, we identify, for the first time, myostatin gene as a target of SIRT6. We demonstrate that under physiologic conditions, SIRT6 negatively regulates Mstn expression via suppressing NF-*κ*B signaling. We also demonstrate a direct link between SIRT6 and the expression of many other intermediary factors implicated in muscle degeneration. Our data show that restoration of SIRT6 expression may serve as a new therapeutic strategy to mitigate muscle wasting associated with chronic diseases.

## Results

### SIRT6 depletion up-regulates muscle atrophy associated genes, whereas, its overexpression maintains myogenic factors

**Figure 1:**
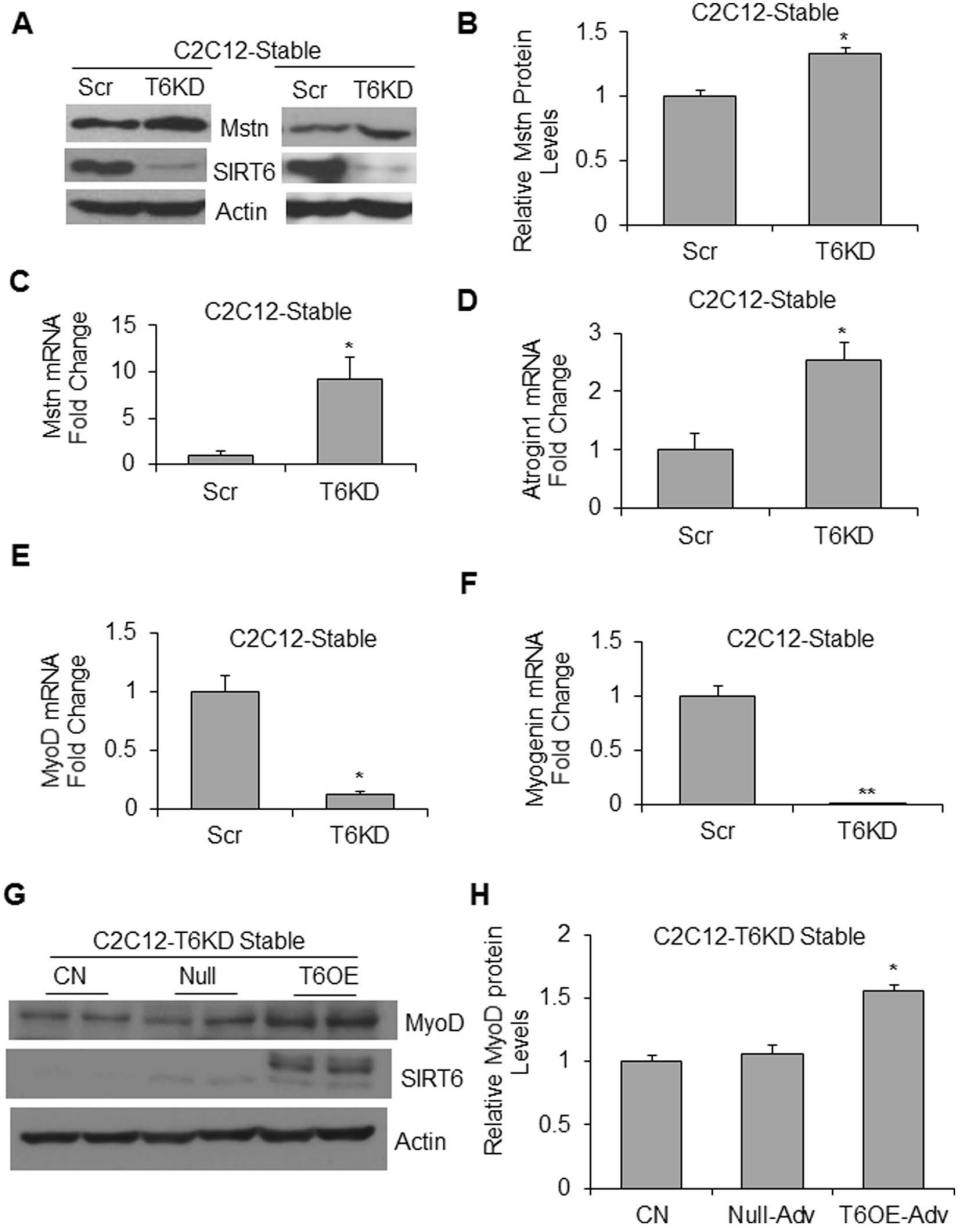
Loss of SIRT6 leads to up-regulation of Myostatin in muscle cells. Mstn protein level (**A,B**) and mRNA expression (**C**) was determined in stable C2C12-scrambled (scr) and SIRT6-shRNA (T6KD) cells by immunoblotting or by qPCR respectively. For both techniques, β-actin expression served as a control. In the same cells, Atrogin1 (**D**), MyoD (**E**) and Myogenin (**F**) mRNA levels were determined by qPCR. (**G**) Stable C2C12-T6KD cells were infected either with empty (Null) or with SIRT6 over-expressing (T6OE) adenovirus, and then differentiated for 72 hours in differentiation medium (DM). DM-0 hour was used as an undifferentiated control (CN). MyoD protein expression was analyzed by immunoblotting **(G)** and quantified (**H**). All bar graphs represent mean ± SEM; n = 3 experiments; *p < 0.05, **p < 0.01.

Myostatin (Mstn) is a master negative regulator of skeletal muscle mass, and its increased expression is considered as a molecular signature of muscle-wasting phenotype^4,25^. Because SIRT6-KO mice display degenerative muscle phenotype, we investigated the effect of SIRT6 depletion on Mstn expression in the mouse myoblast cell line, C2C12. Stable C2C12 cells depleted of SIRT6 were developed by expressing SIRT6-shRNA or control scrambled (scr)-shRNA^26^. The immunoblot analysis revealed significant up-regulation of Mstn in SIRT6 knocked-down (T6KD) cells, compared to controls (Fig. 1A,B). Similar changes were observed for Mstn mRNA levels in SIRT6 depleted cells, indicating transcriptional regulation of Mstn by SIRT6 (Fig. 1C). We then investigated effect of SIRT6 depletion on expression of other genes, previously demonstrated to be associated with muscle wasting. The mRNA expression of Atrogin-1 (MAFbx), a muscle-specific E3 ubiquitin ligase implicated in degradation of sarcomeric proteins^27^, was also increased in SIRT6-KD C2C12 cells (Fig. 1D). Contrary to this, mRNA levels of MyoD and myogenin, two key regulatory factors of myogenic differentiation, were downregulated in SIRT6 depleted cells, compared to controls (Fig. 1E,F). Further, we found that SIRT6 overexpression upregulated MyoD expression in C2C12-T6KD cells (Fig. 1G,H). These data thus demonstrated that SIRT6 exhibits opposite effects on expression of atrophic and myogenic factors, downregulating the former while upregulating the latter group of factors.

**Figure 2:**
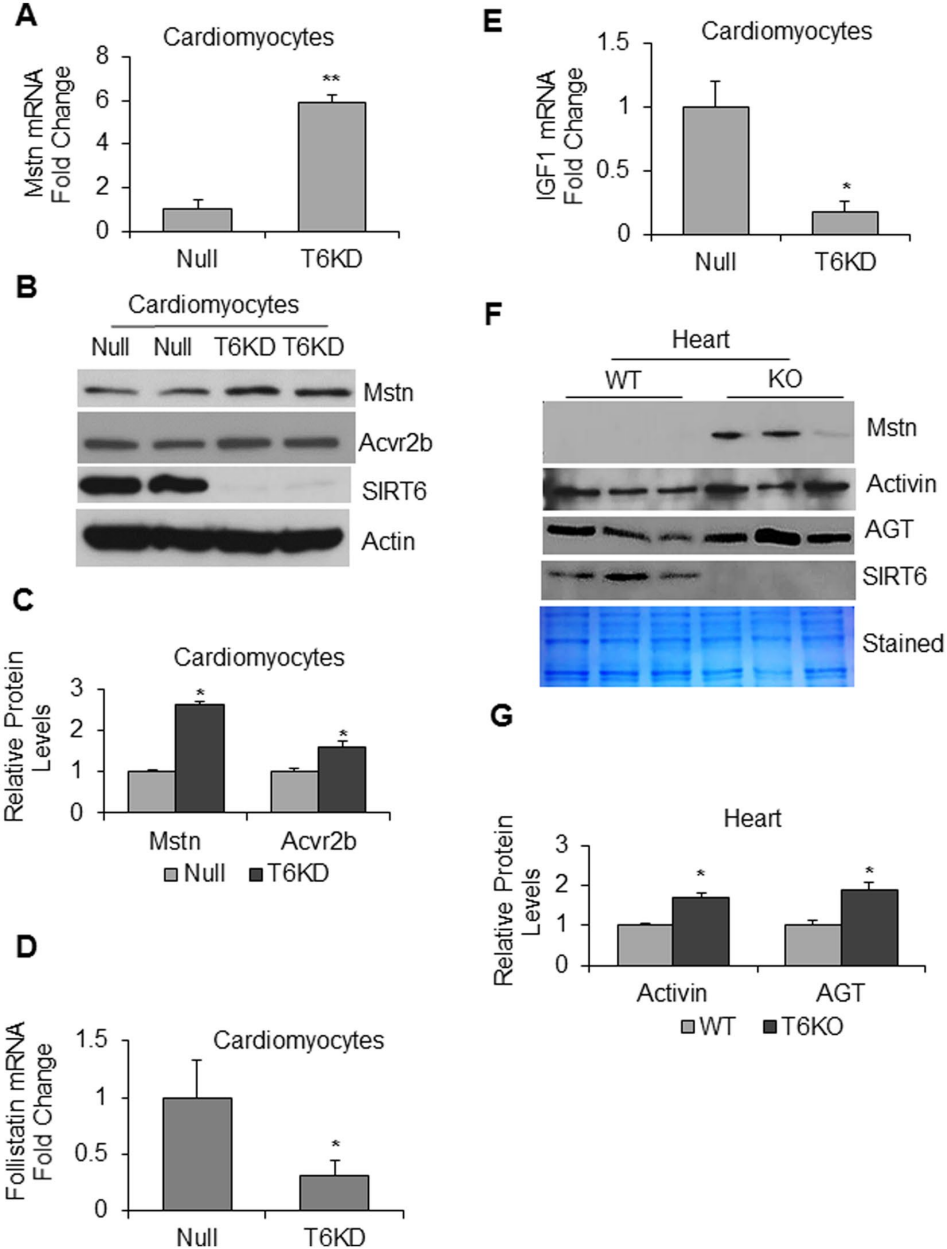
Effect of SIRT6 deficiency on cardiac expression of muscle-atrophy related genes. (**A**) Neonatal rat ventricular cardiomyocytes (cardiomyocytes) were infected with empty adv. (Null) or rat SIRT6-shRNA adenovirus (T6KD) to knockdown SIRT6, and expression of Mstn mRNA was determined by qPCR. (**B**) In the same group of cells, Mstn and Acvr2b expression was determined by immunoblotting. Actin was used as the loading control. (**C**) Quantitation of Mstn and Acvr2b expression in T6KD-cardiomyocytes, compared to Null control. (**D**,**E**) By qPCR, expression of follistatin and IGF1 mRNAs was measured in Null-adv.-infected and T6KD-cardiomyocytes. (**F**) Protein lysates from hearts of wild type (WT) or SIRT6-KO mice were immunoblotted with Mstn, Activin, angiotensinogen (AGT) and SIRT6 antibodies. Coomassie-stained blot was used as loading control. Results of three mouse hearts are shown for each group. (**G**) Quantitation of activin and AGT protein levels in WT and SIRT6-KO hearts. Values represent mean ± SEM for three independent experiments. *p < 0.05, **p < 0.01.

Same as for skeletal muscle, cardiac muscle is also known to develop cachexia^28^. We therefore asked whether SIRT6 depletion would also exhibit similar atrophic changes in cardiomyocytes and heart tissue. The results showed that knocking down of SIRT6 in neonatal rat ventricular myocytes (cardiomyocytes) led to increased expression of both Mstn mRNA (Fig. 2A) and protein levels (Fig. 2B,C). In SIRT6-KD cardiomyocytes we also observed elevated expression of Acvr2b, a high affinity receptor for Mstn as well as activin, the other member of TGF-β superfamily implicated in the muscle degradation pathway^12,29^ (Fig. 2B,C), thus indicating that SIRT6 depletion not only upregulates the atrophic ligands, but also their receptor. Corroborating these findings, we also observed significant reduction in mRNA levels of follistatin and IGF-1, the antagonists of muscle atrophy, in SIRT6-KD cardiomyocytes (Fig. 2D,E). We also analyzed expression of atrophic markers in SIRT6-KO hearts. Same as in isolated cardiomyocytes, we observed significant increase in Mstn expression in SIRT6 deficient hearts, compared to wild type (WT) controls (Fig. 2F). In SIRT6-KO hearts we also found increased levels of activin and angiotensinogen (AGT) (Fig. 2F,G), which are also implicated in development of muscle atrophy^30,31^. Thus these data indicated that, same as in skeletal muscle, SIRT6 deficiency promotes expression of atrophic markers in the heart as well.

**Figure 3:**
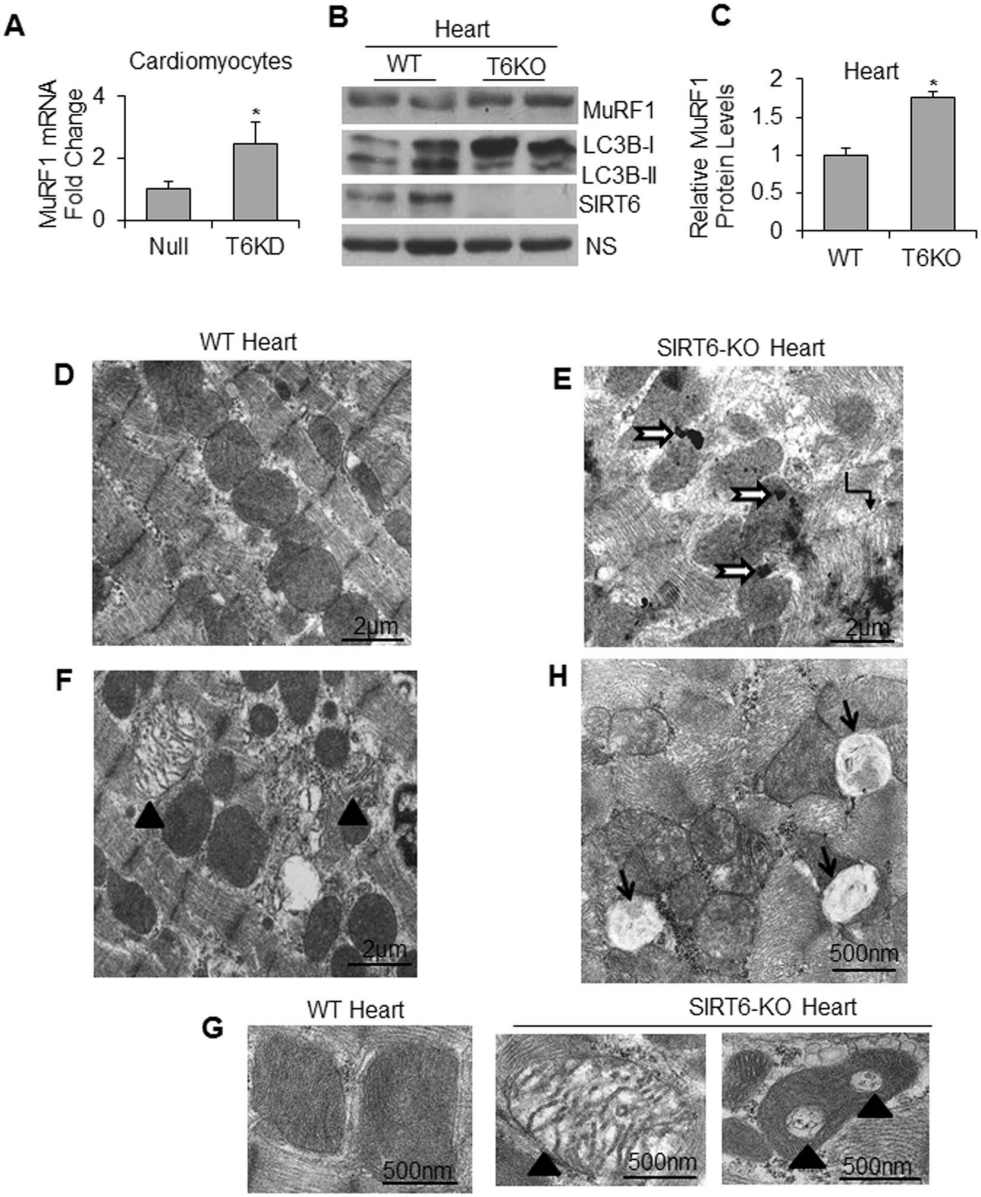
SIRT6 depletion exhibits sarcomeric and mitochondrial deficiencies and up-regulation of factors of muscle degradation pathways. (**A**) MuRF1 mRNA expression was determined by qPCR in Null-adv.-infected and T6KD-cardiomyocytes. (**B**) WT and SIRT6-KO heart lysates were immunoblotted with MuRF1, LC3B and SIRT6 antibodies. A non-specific protein band (NS) used as a loading control. (**C**) Quantitation for MuRF1 protein levels in the heart. Data represented as mean ± SEM for 3–5 samples, *p < 0.05. (**D**–**H**) Representative transmission electron micrographs showing structural integrity of sarcomeres and mitochondria in WT and SIRT6-KO hearts. WT heart section (**D**) shows normal organization of sarcomeres with rows of uniformly dense mitochondria, while SIRT6-KO hearts show (**E**) distorted myofibrils in focal areas (elbow arrow connecter), electron-dense lysosomes (black-bordered white arrows), (F,G right two panels) degenerating mitochondria with reduced electron-dense or vacuolated matrix (black arrowheads) compared to (**G**, left panel) WT and (**H**) accumulation of autophagy-vacuoles (arrows). Scale Bars are as indicated.

The major proteolytic pathways associated with muscle wasting are the ubiquitin-proteasome system (UPS), and the autophagy-lysosome system^32^. In SIRT6-KD cardiomyocytes as well as in SIRT6-KO hearts, we observed augmented levels of muscle ring finger 1 (MuRF1), a muscle enriched E3 ubiquitin ligase implicated as a mediator of muscle atrophy^33^ (Fig. 3A–C). Further, we found upregulation of the autophagic proteolytic pathway in SIRT6-KO hearts as indicated by increased LC3B-I levels (Fig. 3B). While in the WT hearts the ratio of LC3B-I to LC3B-II remained constant, in SIRT6-KO hearts, though LC3B-I increased considerably, LC3B-II (the lipidated isoform) remained mostly unchanged. Consistent with these results, comparative ultrastructural studies of WT and SIRT6-KO hearts revealed loss of myofibrils (Fig. 3D,E) and degenerating mitochondria (Fig. 3F,G) in the absence of SIRT6. We also observed accumulation of autophagic vacuoles containing clearly visible cellular debris in SIRT6-KO hearts (Fig. 3H). These observations are in agreement with the human study where authors reported induction of autophagy, but impaired clearance of autophagosomes in cachectic cancer patients^34^. Together, these results strongly suggest that downregulation of SIRT6 plays a critical role in the development of muscle atrophy.

### Mstn released from SIRT6-deficient cardiomyocytes is functionally active

**Figure 4:**
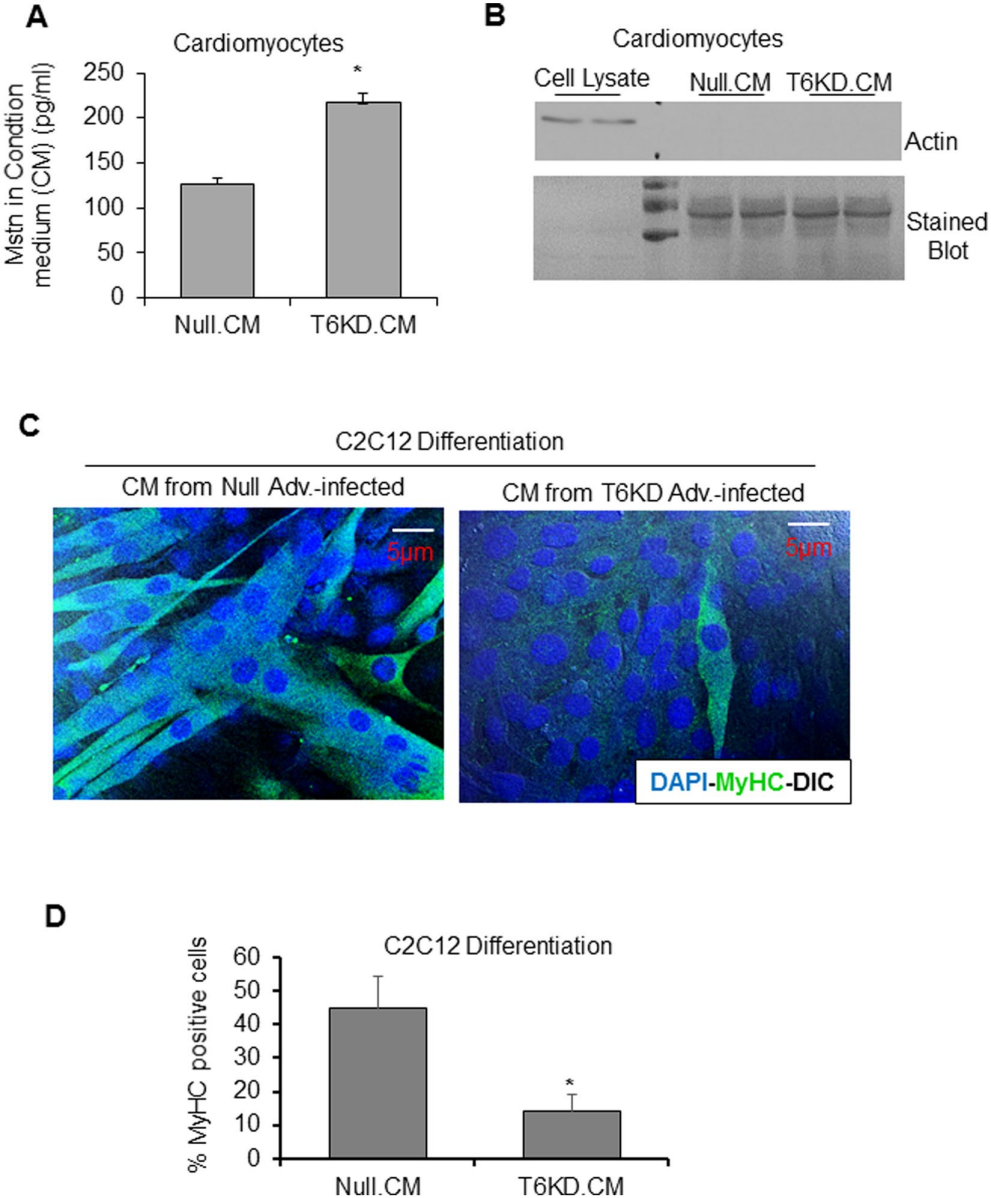
Mstn released from SIRT6-deficient cardiomyocytes is functionally active. (**A**) Secreted Mstn was measured by ELISA in the condition-medium (CM) collected from cardiomyocytes infected with null or SIRT6-KD adenovirus. (**B**) CM harvested as in A was immunoblotted with actin antibody to demonstrate lack of cell lysis. Cell lysate (5 µg) was used to confirm efficacy of actin antibody and blot was stained with coomassie stain to visualize loading of CM. (**C**) Representative confocal images of differentiated C2C12 cells maintained in differentiation medium (DM) + CM from null or T6KD-adenovirus infected cardiomyocytes and immuno-stained with Myosin heavy chain (MF20) antibody (Green). Nuclei were stained with DAPI (Blue) and pictures were merged with DIC to display cell morphology. Scale bars are as indicated. (**D**) Quantitation of data acquired in experiments shown in panel C for myosin heavy chain (MyHC)-positive C2C12 cells. All bar graphs represent mean ± SEM for n = 3 experiments, *p < 0.05.

Compared to skeletal muscle, cardiac expression of Mstn is low, but it is significantly upregulated in failing hearts^5,9^. We found that SIRT6 depletion (Fig. S1) upregulated Mstn, not only intracellularly, but also in the condition medium (CM) as detected by ELISA (Fig. 4A **)**. To demonstrate that Mstn present in CM is secreted from cells, and not resulting from cell death or lysis, we immunoblotted CM for actin, an intracellular protein marker, and found negative results (Fig. 4B). We confirmed efficacy of the actin antibody used by analyzing cell lysates on the same blot. To test whether CM contains proteins, we stained the blot with coomassie blue, and found evidence for the presence of secreted proteins (Fig. 4B). We also confirmed that T6KD for 72 hours does not lead to cardiomyocyte death by flow cytometry using propidium iodide staining as shown in Figs S2A and 2B. We then asked whether the secreted Mstn from cardiomyocytes was active. For this experiment, we incubated C2C12 cells with the CM prepared from SIRT6KD or null-adv infected cardiomyocytes. Cells were immuno-stained using MF20 antibody to detect myosin heavy chain (MyHC) expression (a marker of muscle cell differentiation), and were scored for MyHC-positive cells by confocal imaging. Robust morphology of cells was affirmed by merging DAPI-stained nuclei with the differential interference contrast (DIC) images demarcating cellular boundaries. As seen in Fig. 4C,D, considerably reduced expression of MyHC was observed in C2C12 cells incubated with CM derived from SIRT6-depleted cardiomyocytes, where we detected augmented Mstn, compared to controls. We also used GAPDH staining as an additional cytoplasmic protein-marker to determine health of C2C12 cells, and results indicated that cells were healthy when maintained in SIRT6-KD CM (Fig. S3). These results thus indicated that Mstn released from SIRT6 deficient cardiomyocytes was functionally active.

### Mstn and SIRT6 expression exhibit reciprocal relationship

**Figure 5:**
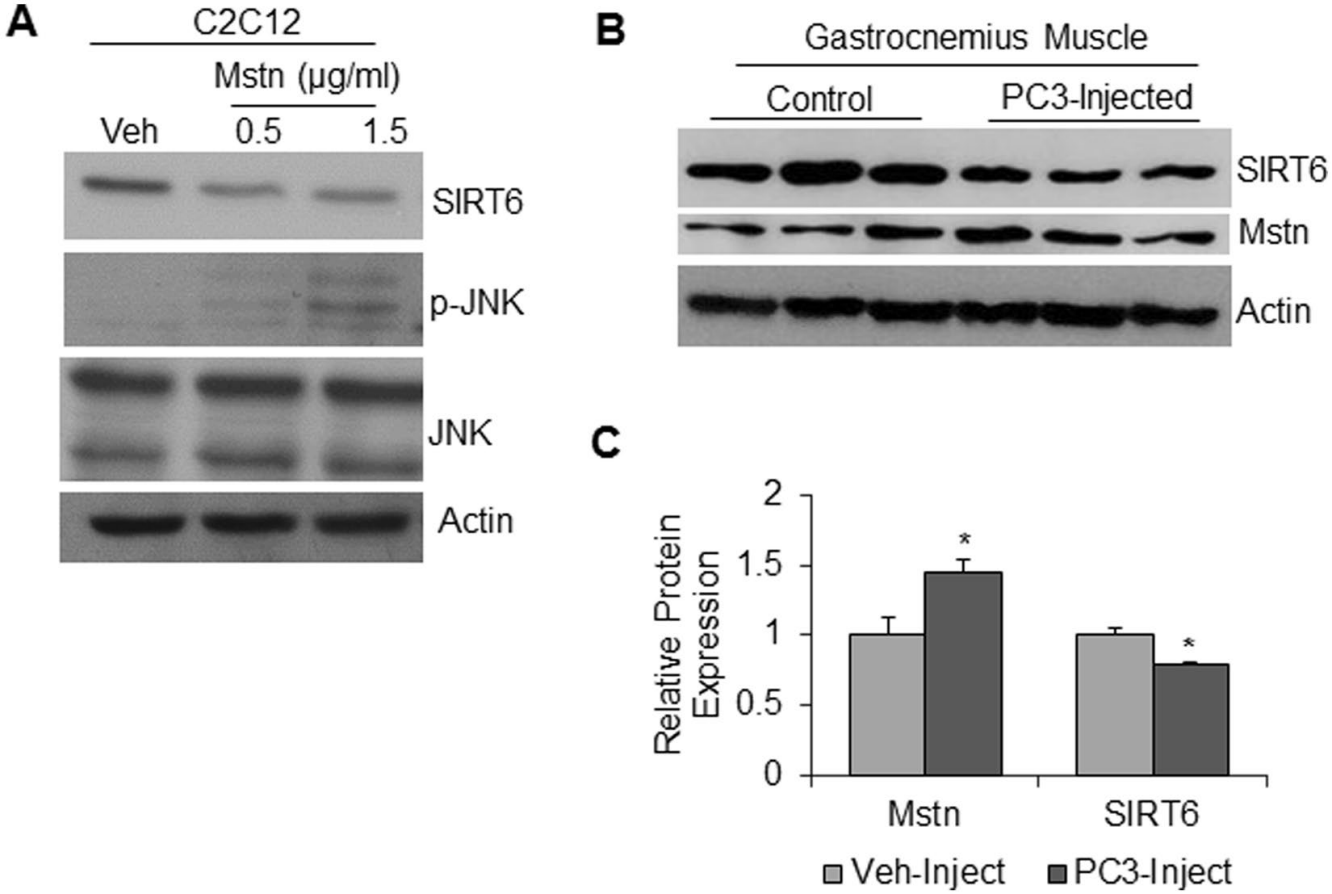
Antagonistic effects of Mstn and SIRT6 on each other. (**A**) Protein lysates from proliferating C2C12 cells treated for 24 hours with recombinant Mstn protein (0.5 or 1.5 µg/ml) were immunoblotted with SIRT6, phospho-JNK and total JNK antibodies. Actin was used as a loading control. (**B**) Representative immuno-blots showing Mstn and SIRT6 expression in gastrocnemius muscles of athymic mice injected with vehicle (control) or prostate cancer (PC3) cells. (**C**) Quantitation of data for the experiment in panel B. n = 5–6 mice, mean ± SEM, *p < 0.05.

Because Mstn is a cytokine released in the blood stream; it exhibits its effect not only locally, but also systemically on other tissues^35^. We therefore asked whether augmented expression of Mstn could cause any effect on intracellular SIRT6 levels. Treatment of C2C12 cells with Mstn caused significant reduction in SIRT6 level (Fig. 5A). To confirm that Mstn treatment was indeed effective in our experiments, we analyzed JNK1 activation in the same samples. JNK1 is known to be activated by Mstn^36^. Increased JNK1 phosphorylation was observed in Mstn treated samples, thus validating activity of the cytokine (Fig. 5A). We also detected a reciprocal correlation between Mstn and SIRT6 in an *in vivo* system. We used a cancer cachectic model, where Mstn expression has been shown to be a causative factor of muscle wasting^37^. We measured SIRT6 and Mstn protein levels in the gastrocnemius muscle of athymic mice injected with PC3 cells (human prostate cancer cell line). We observed when SIRT6 was decreased Mstn was increased in PC3-injected cachectic samples, compared to controls, thus demonstrating a reciprocal relationship between these two factors regulating muscle growth (Fig. 5B,C).

### SIRT6 overexpression mitigates atrophic effect of tumor-induced cytokines in C2C12 cells

**Figure 6:**
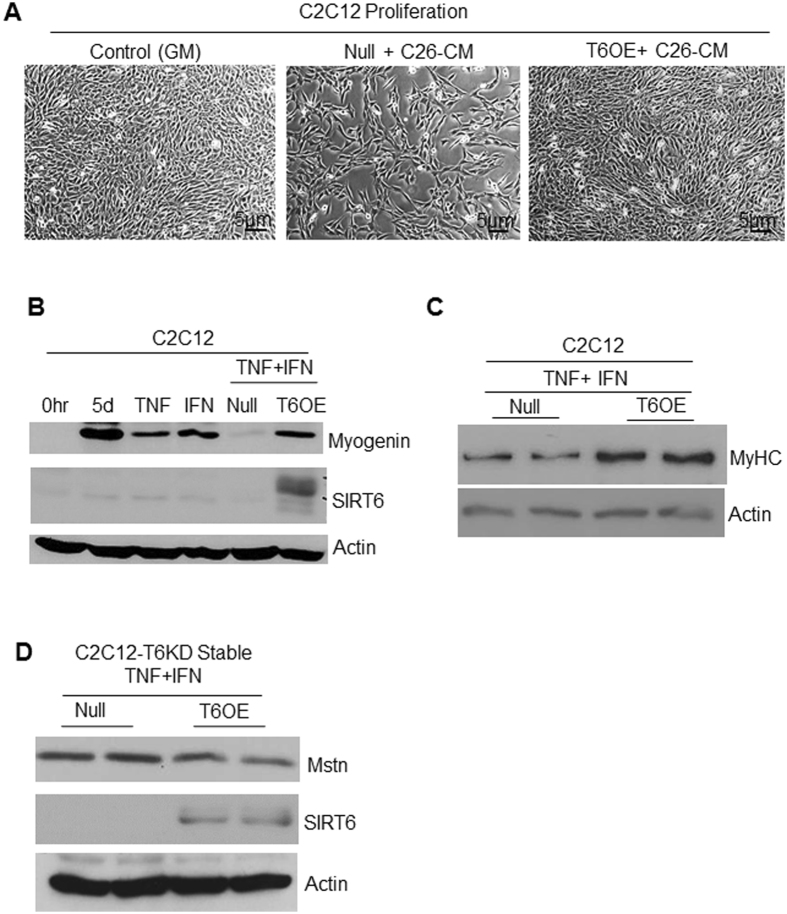
SIRT6 over-expression mitigates atrophic effects of Mstn on muscles cells. (**A**) Phase-contrast images of proliferating C2C12 cells infected with null or SIRT6 over-expressing (T6OE) adenovirus and then treated for 24 hours with C26-condition-medium (CM). CM harvested from C26 colon cancer cells was diluted 1:1 with complete growth medium. Scale bars are as indicated. (**B**) Protein lysates from C2C12 cells differentiated for 3 days in DM, over-expressed with null or SIRT6 over-expressing (T6OE) adenovirus and then treated with TNF-α (20 ng/ml) or IFNγ (100 u/ml) or both as indicated for 48 hours were analyzed by immunoblotting with indicated antibodies. Protein lysates prepared from cells just before exposing them to DM is indicated as zero hour (0 hr). (**C**) Myofilament proteins extracted from C2C12 cells, treated as in panel B, were immunoblotted with MyHC (MF20) and Actin antibodies. (**D**) Protein lysates from stable C2C12-T6KD cells infected with null or SIRT6 over-expressing adenovirus and treated with TNF-α+IFNγ as mentioned above in panel B were immunoblotted with indicated antibodies.

Knowing that SIRT6 downregulation promotes muscle atrophy, we next tested whether restoring SIRT6 expression can overcome the inhibitory effect of tumor-derived cytokines on muscle cell growth. To this end, we used a classical model of cancer cachexia where condition-medium was derived from C26 (colon adenocarcinoma) cells maintained in serum-free DMEM, as a source of tumor-derived cachectic factors. C2C12 cells were either overexpressed with SIRT6 or an empty (null) adenovirus in complete growth medium, and next day proliferating cells were transferred to the C26-condition medium (C26-CM), and cell proliferation was imaged 24 hours later. As seen in Fig. 6A, SIRT6 overexpressing C2C12 cells grew comparable to cells growing in complete growth medium (GM), but null adenovirus infected cells could not overcome the inhibitory effect of C26-CM. This indicated that SIRT6 overexpression could overcome inhibitory effect of cachectic factors derived from tumors on muscle cell growth. In an earlier study, it was demonstrated that pro-inflammatory cytokines, TNFα and IFNγ synergistically downregulate MyHC expression in differentiated C2C12 cells^38^. We employed the same model to determine whether inhibitory effect of these cytokines on C2C12 differentiation can be mitigated by overexpressing SIRT6. We observed that both TNFα and IFNγ, individually as well as in combination, downregulated myogenin expression (Fig. 6B). Interestingly, this inhibitory effect on myogenin was completely blocked by overexpressing SIRT6 in these cytokine-challenged C2C12 myotubes. SIRT6 overexpression also upregulated MyHC expression in these cytokine-exposed C2C12 cells (Fig. 6C). Similarly, when stable C2C12-T6KD cells were overexpressed with SIRT6 and challenged with TNF-α plus IFNγ cytokines, the Mstn expression significantly reduced, compared to controls (Fig. 6D). Taken together, these data indicated that up-regulation of SIRT6 in cachectic conditions helped to block the muscle-wasting state, thus highlighting a new promising role for SIRT6 in prevention of muscle atrophy.

### SIRT6-KO mice show muscle degeneration and fibrosis

**Figure 7:**
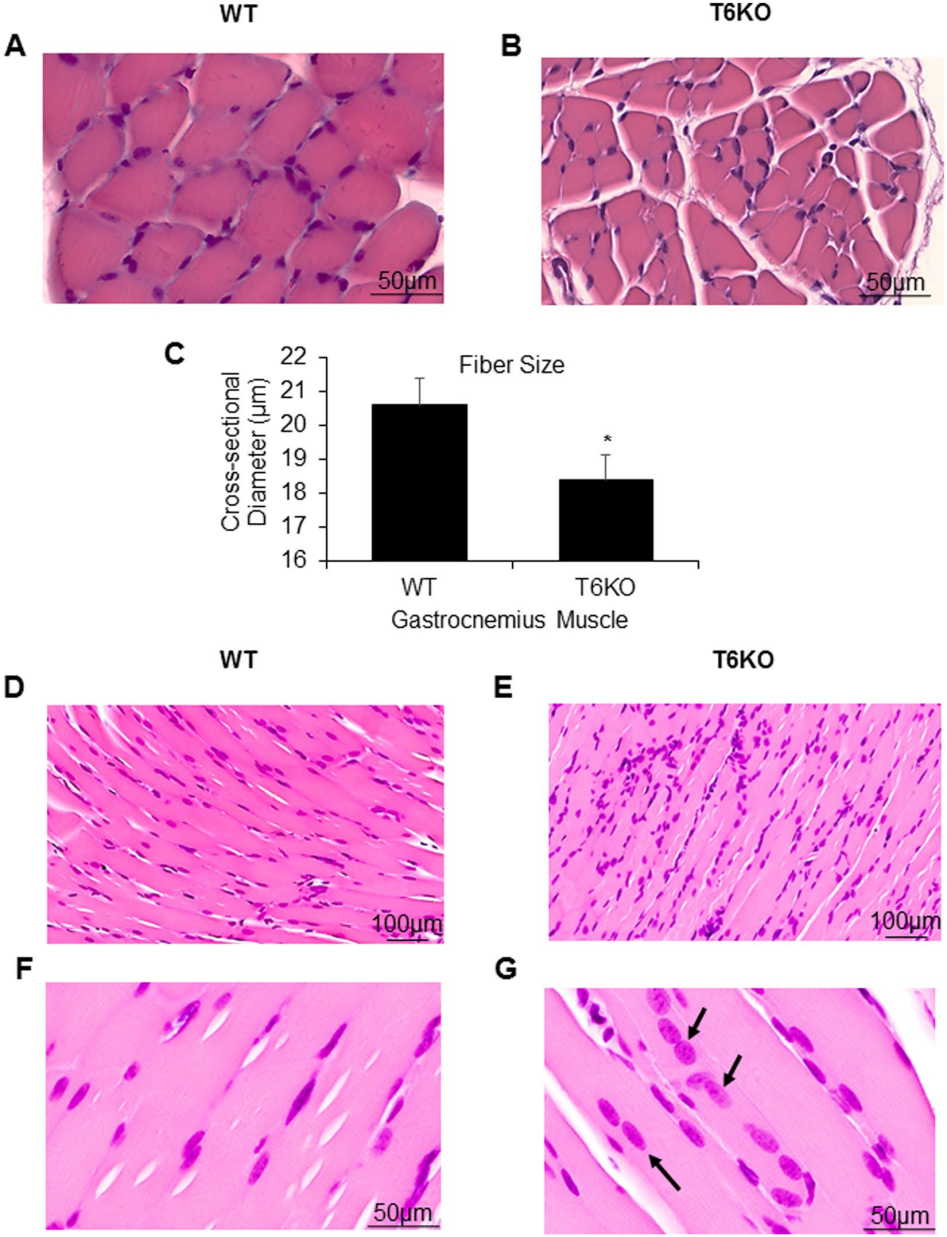
SIRT6-KO mice show degenerative muscle phenotype. Representative sections of gastrocnemius muscles from WT (**A,D,F**) and SIRT6-KO (**B,E,G**) mice stained with hematoxylin-eosin stains to visualize fiber size (**A,B)** and to analyze cross-sectional diameter (**C**) of muscle fibers. (**D, E**) Infiltration of inflammatory cells and (**F,G**) position of nuclei (arrow) in the muscle fibers. Scale bars are as indicated. Data represented as mean ± SEM, n = 3, *p < 0.05.

**Figure 8:**
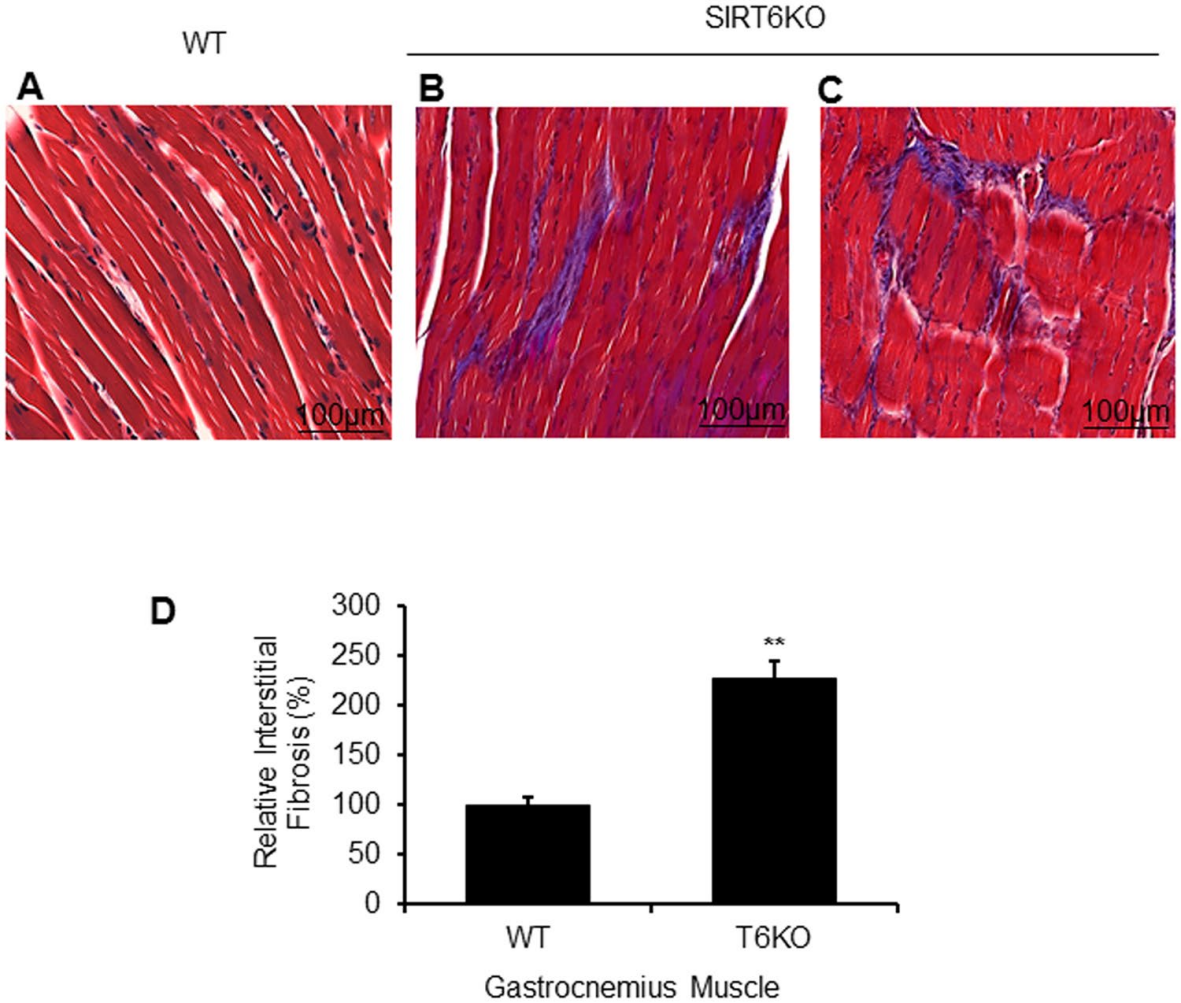
SIRT6-KO mice display increased interstitial fibrosis in gastrocnemius muscles. Masson’s trichrome staining showing collagen deposition (blue) for representative muscle sections from WT (**A**) and SIRT6-KO (**B**,**C**) littermate mice. Scale bars are as indicated. (**D**) Bar graph showing quantitation of fibrosis. Data represented as mean ± SEM for 3–5 mice each, **p < 0.01.

To validate further the role for SIRT6 in regulation of muscle growth, we examined gastrocnemius muscle structure of SIRT6-KO mice. Hematoxylin-eosin stained tissue sections of SIRT6-KO gastrocnemius muscles showed a moderate but significant reduction in fiber cross-sectional diameter, compared to their wild type (WT) littermates (Fig. 7A–C). Also compared to WT (Fig. 7D), in SIRT6-KO muscle sections we noted increased infiltration of inflammatory cells between fibers (Fig. 7E), which is an indicator of diseased muscles. We also observed myofibers with centrally located nuclei in SIRT6-KO muscle, unlike in WT sections where they are located peripherally (Fig. 7F,G). Mis-positioning of myonuclei in the center of myofibers is believed to be a common feature of many muscle pathologies^39,40^. In addition to atrophy, a role for Mstn is also implicated in the development of skeletal muscle fibrosis^41^. Concordantly, we also observed significant fibrosis in the gastrocnemius muscle sections of SIRT6-KO mice compared to WT controls (Fig. 8A–D), consistent with our earlier reports where we had observed development of cardiac fibrosis in SIRT6KO hearts^42^. These results again underscore a role for SIRT6 in maintaining muscle health.

### SIRT6 regulates Mstn expression through NF-*κ*B pathway

**Figure 9:**
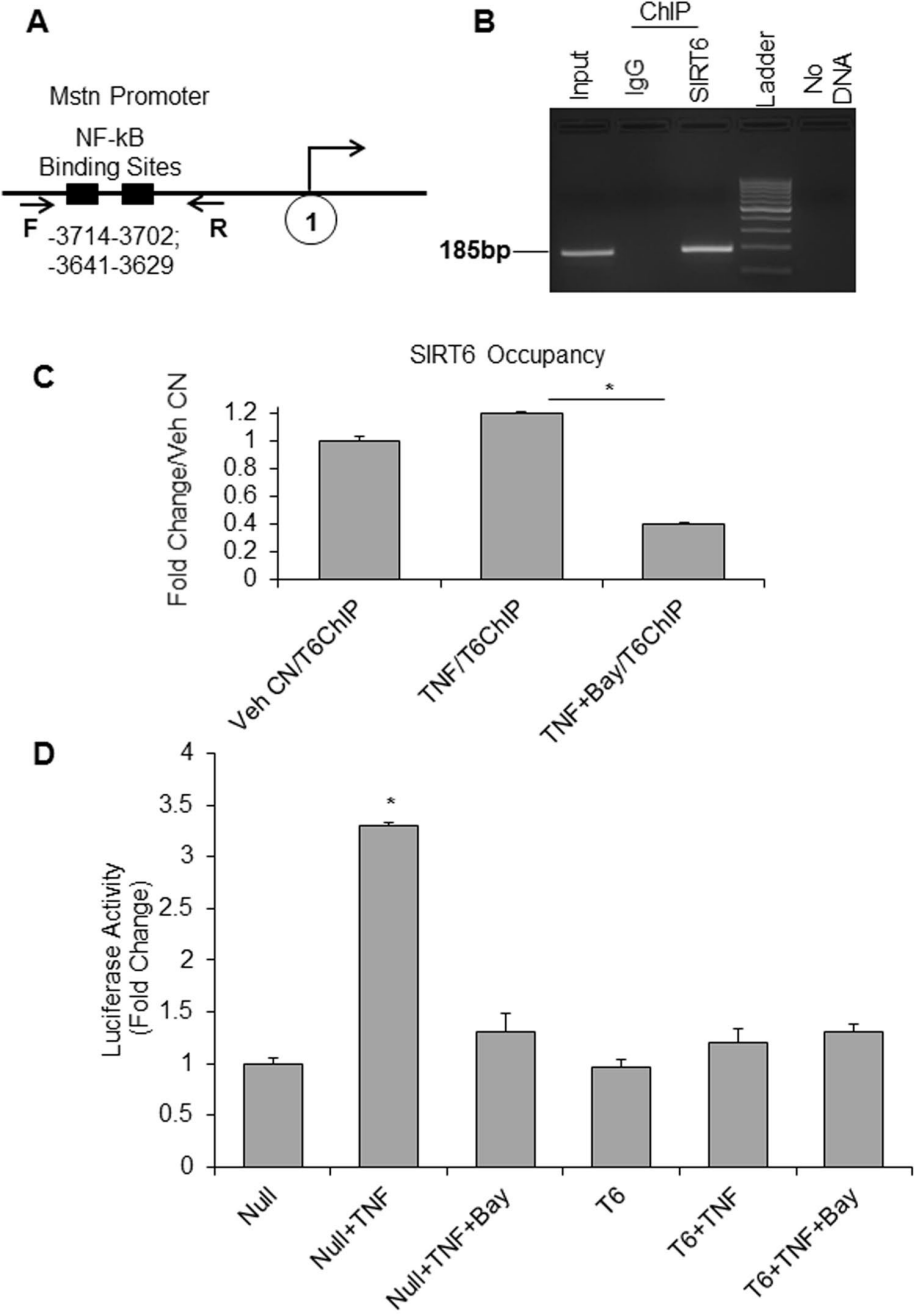
SIRT6 down-regulated Mstn expression via binding to NF-*κ*B sites. (**A**) Schematic representation of mouse Mstn promoter depicting presence of two NF-*κ*B binding sites and the primers (F and R) used for ChIP-qPCR^23^. (**B**) SIRT6 occupancy at Mstn promoter was determined in proliferating C2C12 cells by ChIP assay. The PCR analysis was done for NF-*κ*B sites in Mstn promoter. IgG-ChIP was used as negative control. (**C**) Bar graphs showing relative occupancy for SIRT6 at Mstn promoter as determined by ChIP analysis in C2C12 cells treated with vehicle, TNF-α or TNF-α+Bay11, a NF-*κ*B inhibitor. Data are representative of three independent experiments and shown as mean ± SEM, *p < 0.0001. (**D**) In C2C12 cells transfected with mouse Mstn promoter-Luciferase reporter construct, luciferase reporter assay demonstrates that SIRT6 over-expression inhibits TNF-α-induced myostatin activity by suppressing NF-*κ*B. Data are representative of experiments done in triplicates and shown as mean ± SEM, *p < 0.01.

Several muscle-wasting conditions have been linked to myostatin upregulation^43,44^. Functional role of Mstn in regulation of skeletal muscle growth is well established, and its multiple downstream targets have been identified^6,45,46^. However, there are only handful of its upstream modulators identified so far^13,14,47^. Earlier studies have shown the presence of two consensus sequences for NF-*κ*B binding sites in Mstn promoter^48^ (Fig. 9A). Recently, it was demonstrated that in liver cirrhosis patients, Mstn is transcriptionally up-regulated through activation of the NF-*κ*B-dependent mechanism^23^. There are multiple evidences highlighting importance of NF-*κ*B inhibition for preventing the skeletal muscle waisting^49^. Since SIRT6 has been shown to repress activation of NF-*k*B target genes, we asked whether suppression of Mstn expression by SIRT6 is mediated through blocking of NF-*k*B signaling. The ChIP assay done for C2C12 cells using SIRT6-specific antibody revealed that under basal conditions SIRT6 is bound to Mstn promoter at NF-*k*B binding sites (Fig. 9B). We further confirmed this observation by treating C2C12 cells with TNFα, a known activator of NF-*k*B in skeletal muscle cells^20^ as well as by treating cells with a combination of TNFα and Bay11-7082 (Bay11), a potent inhibitor of NF-*k*B. Bay 11 inhibits NF-*k*B selectively and irreversibly by blocking TNFα-induced phosphorylation of IκB-α^50^. As seen in Fig. 9C, TNFα-mediated increased occupancy of SIRT6 at Mstn promoter was drastically reduced by blocking NF-*k*B activity with Bay11. To further investigate, whether SIRT6 exerts its effect directly through NF-*k*B signaling we transfected a mouse Mstn promoter-luciferase reporter construct in C2C12 cells, and induced the promoter by TNFα treatment. Overexpression of SIRT6 as well as inhibiting NF-*k*B by Bay11 downregulated Mstn promoter/luciferase activity significantly (Fig. 9D). These data thus confirms SIRT6 as a potent repressor of Mstn, and that this action is carried out by blocking activity of the pro-inflammatory transcription factor, NF-*k*B.

## Discussion

**Figure 10:**
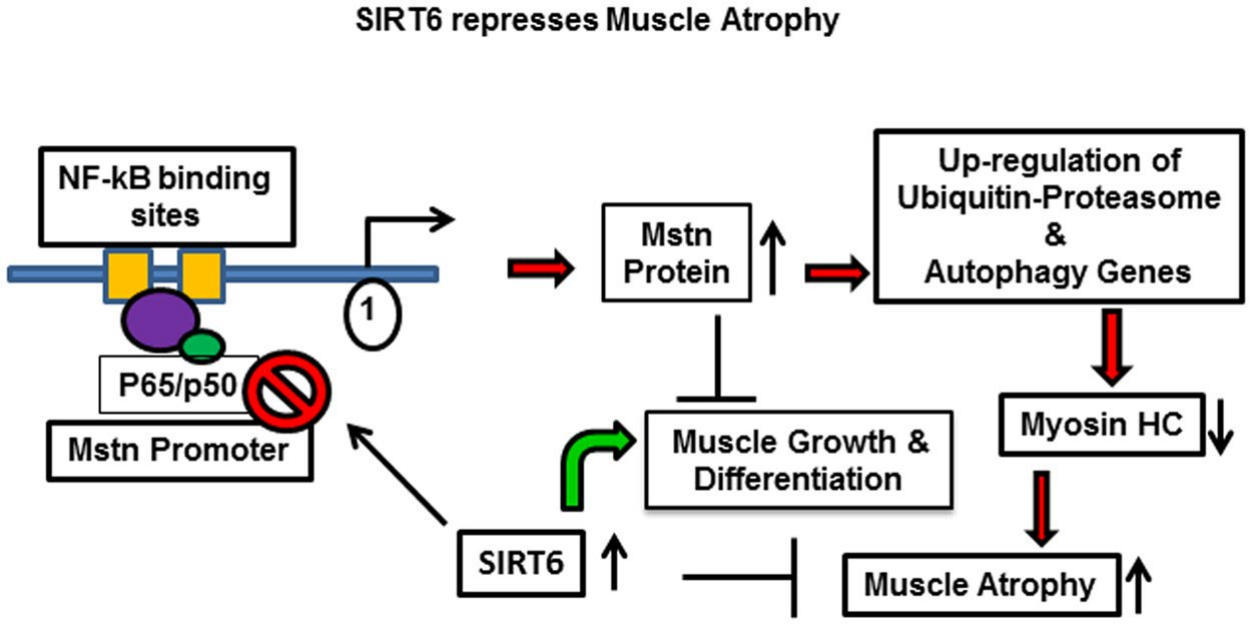
Schematic model illustrating how SIRT6 regulates muscle differentiation and atrophy through NF-*κ*B-Myostatin pathway.

In this study, we report a novel role for SIRT6 as a repressor of Mstn and muscle atrophy. SIRT6 is highly expressed in skeletal muscle tissue^51^ and has been shown to influence metabolic as well as inflammatory pathways^52^. Our data presented here demonstrate that depletion of SIRT6 led to significant up-regulation of Mstn, and its receptor Acvr2b in skeletal muscles. We also observed that SIRT6 depletion activated the protein degradation pathway implicated in muscle atrophy, and this effect was annulled by overexpressing SIRT6. Furthermore, our data demonstrated that the derailed differentiation process of C2C12 cells challenged with pro-inflammatory cytokines, such as TNFα and IFNγ, could be reinstated by overexpressing SIRT6. We also demonstrate that SIRT6 suppressed Mstn expression and muscle atrophic pathways by blocking NF-*k*B signaling as illustrated in the schematic model (Fig. 10).

A dynamic regulation between the anabolic process of protein synthesis and the catabolic process of protein degradation is the key determinant for maintenance of muscle mass and function. In the past decade, considerable efforts have been made to understand the pathophysiology of muscle wasting, however, very little is known about therapeutic strategies pertinent to reversing muscle loss and to regain its function. It has been demonstrated that elevated expression of Mstn is one of the causative steps in the initiation of muscle wasting. In mice with chronic kidney disease, when Mstn was blocked pharmacologically, reduced inflammation, improved insulin/IGF-1 signaling and reduced rate of protein degradation was observed^15^. A cross talk between IGF-1 and Mstn signaling pathways with reference to myogenic differentiation has been reported. Mstn is shown to inhibit IGF-1 signaling, the anabolic arm of muscle protein synthesis^53^, and IGF-1 has been shown to suppress Mstn signaling in skeletal myoblasts^54^, thus these two growth regulatory molecules antagonize each other’s activity. It is noteworthy that SIRT6-KO mice are reported to express low levels of IGF-1^18^, which complements our observation that Mstn is upregulated in these mice.

In a previous study, we have reported hyper-phosphorylation of Akt and induction of cardiac hypertrophy in SIRT6-KO mice^42^. Although development of cardiac hypertrophy and elevated Mstn expression (negative regulator of muscle growth) seem antagonistic, their co-existence have been shown before in models of cardiac hypertrophy. In cardiac-specific myr-Akt over-expressing mice, which develop massive cardiac hypertrophy, up-regulation of Mstn mRNA has been reported^55^. The explanation for increased Mstn expression in these hearts could be related to activation of the negative feedback mechanism to restrain uncontrolled growth of cardiomyocytes. Interestingly, our observation that SIRT6-KO hearts exhibit Akt activation also indicate that muscle atrophy observed in these mice is not resulting from inhibition of pro-growth associated Akt-signaling. Angiotensin-II, another well-known agonist of cardiac hypertrophy is also demonstrated to activate Mstn expression in cardiomyocytes^47^. In the present study, intriguingly we also noted that SIRT6 was downregulated when cells were treated with Mstn, suggesting that Mstn also exerts a negative effect on SIRT6 expression. In an earlier study, Mstn treatment of C2C12 myoblasts was shown to degrade histone acetyl-transferase p300 via activation of the ubiquitin-proteasome system^56^. Whether a similar mechanism operates for degradation of SIRT6 is not known and warrants further investigation.

Inflammatory stimuli are considered important contributory factors for the onset of cachexia. These stimuli function as inhibitors of muscle regeneration and muscle cell differentiation via activating the canonical NF-*k*B pathway^57^. SIRT6 is shown to repress transcriptional activity of NF-*k*B in a tissue- and context-dependent manner. It was demonstrated that upon TNF-α stimulation, nuclear translocation of RELA subunit of NF-*k*B promotes physical interaction between RELA and SIRT6 at the promoters of NF-*k*B target genes^24^, which include many pro-inflammatory cytokines and chemokines^58^. Once recruited to NF-kB binding sites, SIRT6 deacetylates histone H3K9 at these target promoters, which results in compaction of chromatin and termination of NF-*k*B signaling^24^. Another study in vascular endothelial cells reported that SIRT6 deficiency enhances NF-*k*B activity, leading to up-regulation of pro-inflammatory cytokines and this effect could be blocked by re-expression of SIRT6^59^. In addition to playing an anti-inflammatory role by suppressing NF-*k*B signaling, SIRT6 has also been shown to block the c-JUN-dependent expression of pro-inflammatory cytokines like TNF-α and IL-6^60^. SIRT6 also regulates many other pathways in cell- and context-dependent manner. SIRT6 augmentation in cancer cells, but not in normal cells, causes massive apoptosis^61^. Similarly, metabolic effects of SIRT6 appear to vary in different tissues. While SIRT6 depletion promotes glucose uptake in skeletal muscle, similar change in glucose metabolism not noted in the heart, liver or brain tissues^62^. Additionally, SIRT6-mediated activation of NRF2 was found only for human mesenchymal stem cells, but not for vascular endothelial cells^63^. Another study has reported that over-expression of SIRT6 in different tissues of mice, either increases or decreases p16INK4a, a widely recognized biomarker for cellular senescence^64^. Therefore, taking into account the pleotropic effects of SIRT6, our data presented here point to an anti-inflammatory role for SIRT6 both in skeletal and cardiac muscle cells.

The antagonist effect of SIRT6 on Mstn is also supported by many parallel findings reported for the mode of action for these two molecules. SIRT6-KO mice not only exhibit cachectic phenotype, but also show loss of subcutaneous fat and deficiency in bone mineral density, resonating to an osteoporotic state. These mice suffer from osteopenia with 30% reduction in bone mineral density (BMD)^18^. For normal osteogenic differentiation of mesenchymal stem cells, SIRT6 was shown to play an essential role through suppression of NF-*k*B signaling^65^. Similarly, myostatin inhibition was reported to improve muscle mass and bone density^66^. In a human clinical trial, use of an Mstn inhibitor resulted in significant improvement in bone mass as well as in BMD by 3.4%^67^. Results reported from Mstn-KO mice displaying resistance to diet-induced obesity^68^ are also in parallel to SIRT6 overexpressing mice, which are protected from developing high-fat diet-induced metabolic syndrome^69^. Furthermore, there are commonalities between function of SIRT6 and Mstn in their gender-specific effects. Muscle-specific Mstn overexpression resulted in 20% muscle atrophy only in males, but not in females^70^. Similarly, SIRT6 overexpression resulted increased life-span in male mice, but not in females^71^. Moreover, sexual dimorphism was observed in cardiac atrophy exhibited by tumor-bearing mice, where male mice carrying tumor burden displayed severe loss of cardiac mass and heightened pro-inflammatory response, compared to their female counterparts^28^. These reports together support a reciprocal relationship between SIRT6 and Mstn, as was observed in this our study.

Dysregulation of skeletal muscle metabolism is another contributory factor for loss of muscle mass and inability to regenerate. SIRT6 is a key regulator of metabolic homeostasis. SIRT6 not only controls glycolysis and gluconeogenesis, but also fat homeostasis and obesity^72^. SIRT6-KO mice suffer with severe hypoglycemia, which is associated with enhanced rate of glucose uptake by skeletal muscle^26,62^. One contributory factor for this effect could be increased Mstn levels we observed in these mice. Mstn was shown to increase basal glucose uptake in human placental tissues^73^. In SIRT6 transgenic mice, moderate overexpression of SIRT6 enhanced insulin-sensitivity in skeletal muscles and liver. These mice also showed improved glucose metabolism despite feeding with high-calorie diet^74^. Another study has demonstrated that high-fat diet fed SIRT6 overexpressing mice were protected from impaired glucose tolerance and fat accumulation^69^. Along the same lines, increased glucose utilization and enhanced insulin sensitivity was reported for whole body Mstn-KO mice, as well as for conditional mice, where Mstn signaling was inhibited in skeletal muscle^75^. These reports again support our proposition that suppression of Mstn by SIRT6 may contribute to improved insulin sensitivity and glucose homeostasis, and thereby overall muscle health.

Besides chronic diseases, skeletal muscle loss also occurs during aging and from disuse. Such a muscle loss could be due to reduction in number of muscle fibers or their size leading to decline in force generation and muscle strength. Cachexia and sarcopenia (muscle loss with aging) both are devastating conditions since muscle strength is not easily rectifiable, worsening the underlying cause. As of 2013, approximately 9 million patients, that is 1% of all patients carrying any chronic disease, were estimated to be affected by cachexia^3,76^, and ironically, this is a largely underestimated syndrome. More than 5 million people in the United States alone present with cachexia stemming from some disease. With increasing aging population adding further to these numbers, it is especially more important to identify additional players in the pathways contributing to muscle loss. In this study, we unravel SIRT6 as an endogenous repressor of the muscle atrophic pathway, which opens a new therapeutic option for treating this devastating muscle wasting disease.

## Material and Methods

### Antibodies and other reagents

Antibodies and conjugates used in this study are listed in Table 1. Empty (Null, #1240) and human SIRT6 overexpressing (#1556) adenoviruses were purchased from Vector Biolabs. Rat-specific SIRT6-shRNA adenovirus was made by Welgen Inc. (Worcester, MA, USA). Mammalian protease and phosphatase inhibitors, mouse TNFα were purchased from Sigma. Recombinant mouse IFNγ and recombinant GDF8/myostatin were purchased from R&D systems. Mouse wild-type myostatin promoter cloned in Luciferase reporter plasmid pGL4.76 was a kind gift from Dr. Srinivasan Dasarathy, Cleveland clinic, Cleveland, Ohio.

**Table 1:** Antibodies used in this study.

Antibody Name	Company	Catalog Number
Myostatin	R&D Systems	MAB788
Angiotensinogen	R&D Systems	MAB6966
Myosin heavy Chain	R&D Systems	MAB4470
Activin	R&D Systems	AF338
SIRT6	Cell Signaling	12486
Phospho-JNK	Cell Signaling	9251
JNK	Cell Signaling	9252
Myogenin	Santa Cruz Biotechnology	sc-12732, sc-576
MyoD	Santa Cruz Biotechnology	sc-760
Actin	Santa Cruz Biotechnology	sc-1616
GAPDH	Santa Cruz Biotechnology	sc-25778
MuRF1	Abcam Inc	ab172479
Acvr Type 2b	Abcam Inc	ab180185
LC3B	Sigma	L7543
Anti-rabbit HRP conjugate	Santa Cruz Biotechnology	sc-2077
Anti-mouse HRP conjugate	Santa Cruz Biotechnology	sc-2096
Anti-rat HRP conjugate	Santa Cruz Biotechnology	sc-2065
Anti-goat HRP conjugate	Santa Cruz Biotechnology	sc-2056
βActin-HRP	Santa Cruz Biotechnology	sc-47778-HRP
Anti-mouse AF488 conjugate	Invitrogen	A21202
Anti-rabbit AF594 conjugate	Invitrogen	A21207

### Cell culture studies

Primary cultures for neonatal rat ventricular cardiomyocytes were generated using established method^77^. Briefly, cardiomyocytes were isolated from 1–3 day old Sprague-Dawley rat pups by digesting the minced heart ventricular tissue with collagenase type II enzyme (Worthington, NJ, USA) 6–7 times at 37 °C in a shaker water-bath. Cells were pelleted down, re-suspended in DMEM-5% FBS and pre-plated for one hour to remove attached fibroblasts. Unattached cardiomyocytes from the suspension were pelleted, re-suspended in PC-1 medium (Lonza, Germany), plated on fibronectin-coated (25 µg/ml) tissue culture plates (200,000 cells/cm^2^) and maintained in PC-1 medium. After 24 hours, unattached cells were aspirated out and PC-1 medium was replenished. Next day cells were infected with either empty (Null) or SIRT6-shRNA (T6KD) or SIRT6 over-expressing (T6OE) adenoviruses at 100 multiplicity of infection (MOI). For SIRT6 knock-down (and the corresponding Null-control) cells were harvested 72 hours after infection. Medium from adenovirus-infected cardiomyocytes (Null or T6KD CM) was collected, centrifuged at 1000 rpm for 5 minutes and then passed through 0.22 µ syringe filter. We used ELISA (R&D systems, USA) to estimate secreted Mstn concentration in these condition media (CM). To study effect of CM on differentiation of C2C12 cells, CM was concentrated ten-fold using Amicon ultra-15 centrifugal filters (Millipore, USA) as per the manufacturer’s protocol. C2C12 cells (between passage 15–20) were plated in DMEM with 1% penicillin-streptomycin (PS) and 10% FBS (growth medium). For studies with differentiated cells, 80% confluent cultures of C2C12 cells were maintained in differentiation medium (DM) [DMEM with 1% PS, insulin (10 µg/ml) and 2% heat-inactivated horse serum (Sigma, USA) instead of 10% FBS] for 3–5 days. Adenoviral infections were done as above. Treatment of C2C12 cells with TNFα (20ng/ml), IFNγ (100 u/ml) was done in DM. Myostatin treatment of proliferating C2C12 cells was done in 1%FBS containing DMEM. The NF-*κ*B inhibitor Bay11-7082 (Santa Cruz Biotech, USA) was used at a concentration 2 µM in DMEM with 5% FBS for 24 hours. Stable C2C12-scrambled-shRNA (C2C12-scr) or SIRT6-shRNA (C2C12-T6KD) cell lines were a kind gift from Dr. Chu-Xia Deng, NIH, USA. Dr. Denis Guttridge from the Ohio state University, Columbus, Ohio, USA, provided colon adenocarcinoma (C26) cell line. C26 cells were grown over-night to 70% confluency in complete growth medium and then after washing twice with PBS maintained further for 24 hours in serum-free DMEM. This medium was harvested, centrifuged and passed through 0.22 µ syringe filter. This C26-CM was diluted 1:1 with complete growth medium and was used to check effect on C2C12 proliferation.

### Animal studies

SIRT6 WT and KO mice (Jackson Laboratory, stock number 006050) were on 129svJ background^18^. Tumor model of cachexia was generated by injecting one million proliferating PC3 tumor cells reconstituted in matrigel matrix (Corning, USA), subcutaneously into the flanks of 6-week old athymic BALB/c (Envigo, USA) male mice. After 45 days, control (vehicle-injected) and tumor cells-injected mice were euthanized and gastrocnemius muscle tissue from un-injected flank was harvested. All animal protocols were reviewed and approved by the University of Chicago Institutional Animal Care and Use Committee. All methods were performed in accordance with the relevant guidelines and regulations of the Biosafety Committee of the University of Chicago.

### ELISA for Mstn

To estimate secreted Mstn in the condition medium from cardiomyocytes infected with either null or SIRT6-shRNA (T6KD) adenovirus, we used quantikine GDF8/Myostatin immunoassay ELISA kit (R&D systems, MN, USA) and followed the manufacturer’s instructions. PC-1 medium used for maintaining cardiomyocytes was free of FBS.

### Cell death assay

Cardiomyocytes were cultured in six-well plates and were infected with either null or T6KD adenovirus at 100 MOI for 72 hours. Cells were trypsinized, pelleted at 4 °C and washed twice with PBS. Cells were then resuspended in 400 µl PBS containing 2% FBS and propidium iodide (0.5 µg/ml) and analyzed by flow cytometry using BD LSR II (Becton-Dickinson, San Jose, CA). Results were processed using FlowJo software.

### Luciferase reporter assay

C2C12 myoblasts were transfected with pGL4.76 luciferase reporter plasmid containing mouse myostatin full-length promoter using Lipofectamine 2000 reagent (Invitrogen) followed by infection with either null or SIRT6 over-expressing adenovirus at 100 MOI. Cells were then differentiated for 48 hours and were treated with either TNFα (20 ng/ml) or TNFα+Bay11-7082 (2 µm). Luciferase activity was estimated using luciferase assay system (Promega) and normalized to protein concentration.

### RNA extraction and real-time RT-PCR (qPCR)

Total RNA was extracted from cardiomyocytes and other cell lines using TRIzol reagent (Invitrogen, CA, USA) as per manufacturer’s instructions. RNA preparations were treated with DNase I (Qiagen, MD, USA) and purified by phenol:chloroform extraction and by using phase-lock gel columns (5 Prime, VWR, USA) as per manufacturer’s instructions. For cDNA synthesis, 1–2  µg of purified RNA was reverse transcribed using RevertAid First strand cDNA synthesis kit (Fermentas, USA) and resultant cDNA was diluted 10-fold before using for qPCRs. PCRs were performed using SYBR Green kit (Thermo-Fisher, USA). Primer sequences are as tabulated in Table 2. For normalization, β-actin was used as the endogenous control and fold-changes in gene expression were determined using comparative threshold cycle (2^−∆∆Ct^) method^78^.

**Table 2:** Primers used for real-time PCR: m: mouse, r: rat.

Gene	Sequence (5′-3′)
mMstn-F	CAGCCTGAATCCAACTTAGG
mMstn-R	TCGCAGTCAAGCCCAAAGTC
mMyogenin-F	GCTCAGCTCCCTCAACCAG
mMyogenin-R	ATGTGAATGGGGAGTGGGGA
mMyoD-F	GATGGCATGATGGATTACAGC
mMyoD-R	GACTATGTCCTTTCTTTGGGG
mAtrogin1-F	AACATGTGGGTGTATCGG
mAtrogin1-R	TCTTGAGGGGAAAGTGAG
mβ-Actin-F	TTCTTTGCAGCTCCTTCGTTGCCG
mβ-Actin-R	TGGATGGCTACGTACATGGCTGGG
rMstn-F	AGAGAGAGGCGAATGTGGAA
rMstn-R	TCACTGCTGTCATCCCTCTG
rMuRF1-F	TGTTCTGGTAGGTCGTTTCCG
rMuRF1-R	ATGCCGGTCCATGATCACTT
rFollistatin-F	AATGCCTACTGTGTGACCTGT
rFollistatin-R	GGCTCATCCGACTTACTGT
rIGF1-F	GGGAGACAACCTTGTCAAGCACCTA
rIGF1-R	GAAATGAATGCCTGCTGAGGT
rSIRT6-F	TTTATTGTTCCCGTGCGGCG
rSIRT6-R	GGGTCGAATATCTCGGGCAG
rβ-Actin-F	GACTTCGAGCAAGAGATGGCCACT
rβ-Actin-R	AGCACTGTGTTGGCATAGAG

### Histology, Immunostaining and Immunoblotting

Gastrocnemius muscle tissues from mice were fixed in neutral formalin. Tissue sections were processed at the HTRC core facility of the University of Chicago. Sections were stained with hematoxylin-eosin stains for assessing muscle structure and with Masson’s trichrome stain for fibrosis. Imaging of stained histological sections was done using Perkin Elmer’s Pannoramic Scan whole slide scanner with Pannoramic viewer software (3dhistec Ltd., USA), and quantitation was done using ImageJ. Phase-contrast images of C2C12 cells were acquired at 4x using Nikon phase contrast-2 microscope (Japan) equipped with MicroFire Optronics camera. To determine the effect of condition medium on extent of myosin heavy chain expression in C2C12 cells, condition medium harvested from cardiomyocytes was concentrated as explained above. C2C12 cells were maintained in DM+concentrated condition medium (1:1) and horse serum adjusted to a final concentration of 2%. Medium was changed every day. After three days, cells were immuno-stained with primary antibodies for myosin heavy chain (mouse MF20 antibody) and rabbit GAPDH. Secondary antibodies used were anti-mouse Alexa Fluor 488- and anti-rabbit Alexa Fluor-594-conjugated. Immunostaining was done as described previously^79^. Immuno-stained slides were imaged using Leica TCS AOBS SP2 laser scanning confocal microscope running the Leica LCS software. Protein lysates from heart ventricles, gastrocnemius muscles or cells were prepared in radio-immunoprecipitation assay (RIPA) buffer (50 mM Tris.HCl, pH 7.5, 300 mM NaCl, 0.1% Nonidet P-40, 1% Triton X-100, 1 mM EDTA, protease and phosphatase inhibitors). Typically, 20–30 µg of protein lysates were used for immunoblotting. Myofilament proteins were extracted using a proteo-extract subcellular proteome extraction kit (EMD Millipore, USA) as per manufacturer’s instructions. Immunoblots were quantitated using Quantity one software (BioRad).

### Electron Microscopy for heart samples

Portion of a heart tissue was harvested from the mice, fixed in the EM fixative as per the standard protocol and processed further at the transmission electron microscope core facility of the University of Chicago. Images were captured at 300 kV with FEI Tecnai F30 electron microscope equipped with a high-performance Gatan charge-coupled device (CCD) camera.

### ChIP Assay

Chromatin immunoprecipitation (ChIP) was performed on 70–80% confluent C2C12 cells grown in growth medium. Cells were treated with either vehicle (4 mM HCl containing 1 mg/ml BSA+DMSO) or TNF-α or TNF-α+Bay11-7082 in DMEM/5%FBS for 24 hours as described above and then crosslinked using 37% formaldehyde (Thermo-Fisher Scientific) at a final concentration of 1% in the medium for 10 minutes at room temperature on rocking platform. Cells were washed with ice-cold 1XPBS briefly and cross-linking reaction was stopped by adding glycine at a final concentration of 125 mM to the medium and rocking plates for 5 min at room temperature. Cells were washed again with ice-cold 1XPBS briefly and harvested in cold PBS. Pelleted cells (2500 g, 10 minutes at 4 °C) were lysed in 500 µl SDS-lysis buffer (1% SDS, 10 mM EDTA, 50 mM Tris.HCl, pH 8.0) containing protease inhibitors. DNA sonication was carried out at 4 °C using Misonix S-4000 sonicator equipped with the micro-tip (Qsonica LLC, CT, USA). Conditions used for sonication were 30 cycles (20 sec on, 1 minute off) at an amplitude of 40. Samples were centrifuged at 13,000 g for 10 minutes at 4 °C and supernatant was used for ChIP assay. An aliquot of each supernatant was kept aside as input control. 5–20 µg of sonicated DNA was used for immunoprecipitation with either rabbit anti-SIRT6 antibody (Abcam ab62739) or with rabbit IgG (Diagenode, NJ, USA) for 4 hours on rotator at 4 °C. Washing of immuno-complexes and further processing of all the samples was done using ChIP-IT Express kit (Active Motif, CA, USA) as per the manufacturer’s instructions. SIRT6 binding to the Mstn promoter at NF-*κ*B sites was tested using either standard or real-time qPCR. Primers^23^ used for the ChIP-PCR were; Forward: 5′-CTG GCA AGT CTG AGC ATC TG-3′ and Reverse: 5′-CAG AGG GAG AGA AGG TTA GGA A-3′. Fold enrichment at the promoter was calculated using the standard method described by the same kit’s manufacturer.

### Data availability

All data generated or analyzed during this study are included in this article. All full-length blots are presented in the supplementary Figure.

### Statistical Analysis

All graphical data is presented as mean ± standard error (SEM). Statistical difference between two groups was determined using two-tailed paired *t* test.

## Electronic supplementary material

Supplementary Information
