# Supplementary material for: The histone deacetylase SIRT6 blocks myostatin expression and development of muscle atrophy
Source: Sci Rep. 2017 Sep 19;7:11877. doi: 10.1038/s41598-017-10838-5 (PMC5605688; doi:10.1038/s41598-017-10838-5)

Supplementary Information

Title: The histone deacetylase SIRT6 blocks myostatin expression and development of muscle atrophy

Authors: Sadhana A. Samant, Abhinav Kanwal, Vinodkumar Pillai, Riyue Bao and Mahesh P. Gupta

Figure S1

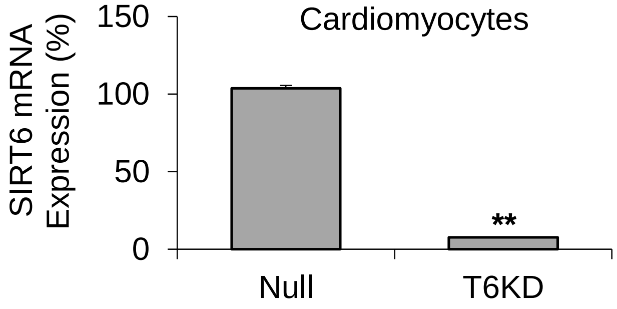

Figure S1: Validation of SIRT6 knockdown by qPCR in cardiomyocytes infected with Null or rat SIRT6-shRNA (T6KD) adenovirus. Bar graph represents mean ± SEM for n=3 experiments, ** $p<0.001$.

Figure S2

A

B

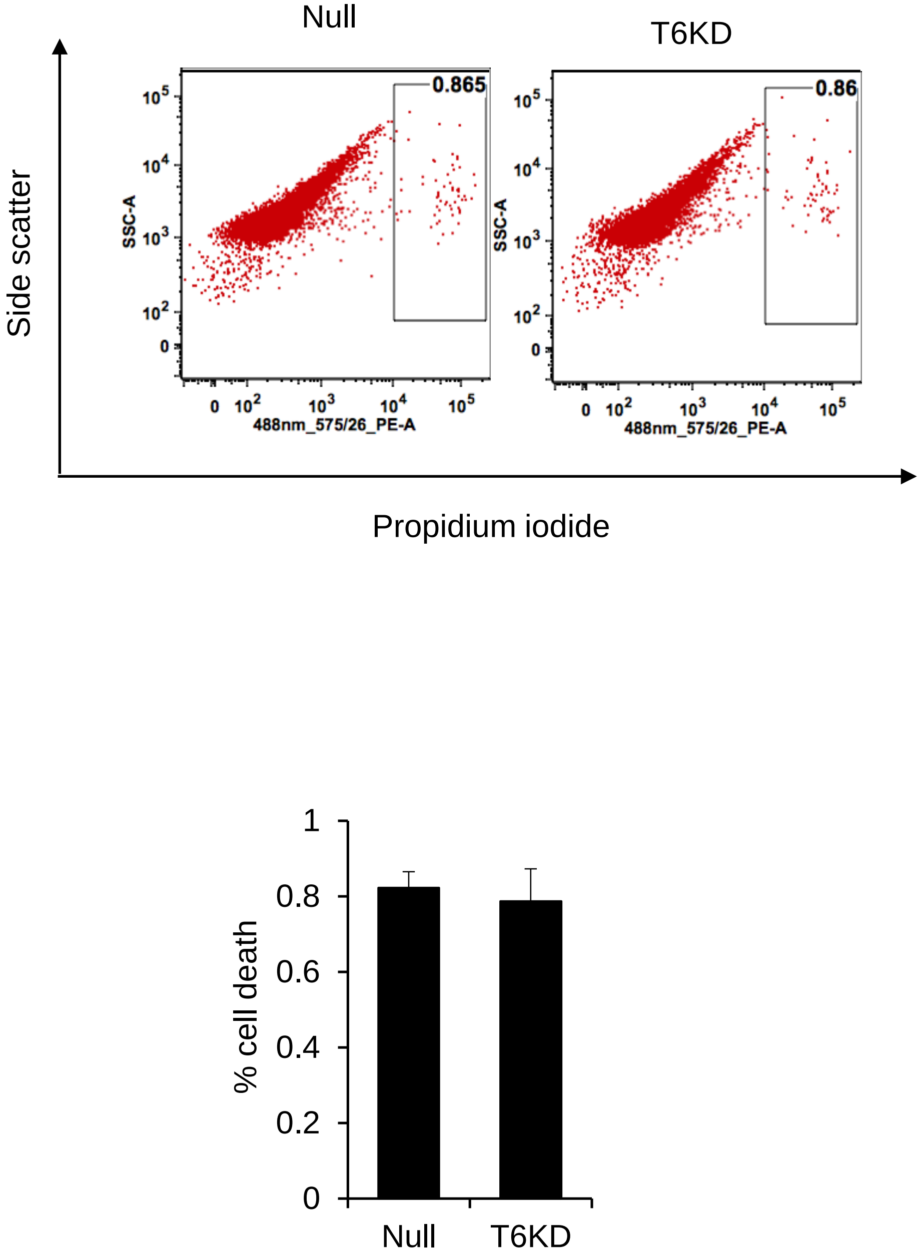

(A) Cardiomyocytes were infected with either Null or rat SIRT6-shRNA adenovirus (T6KD) and after 72 h of infection, cells were harvested and extent of apoptosis was measured by estimating the percentage of propidium iodide positive cells by FACS analysis. (B) Quantification of cell death in different groups of cells. All values are means ± SEM; *n* = 7

Figure S3

C2C12 Differentiation

CM from Null Adv.-infected

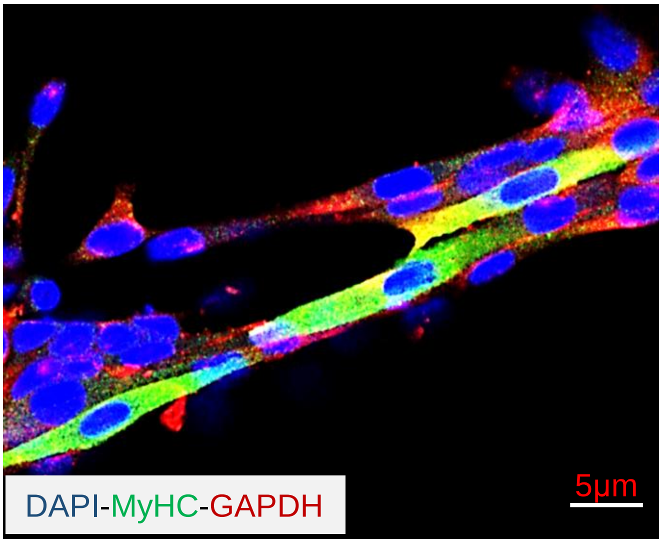

CM from T6KD Adv.-infected

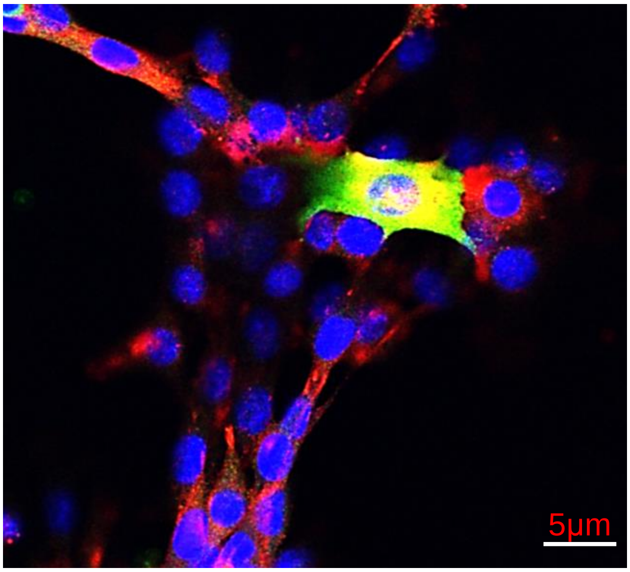

Representative confocal images of differentiated C2C12 cells maintained in differentiation medium (DM) + CM from null or T6KD-adenovirus infected cardiomyocytes and immuno-stained for Myosin heavy chain (MF20) (Green) and GAPDH (Red). Nuclei were stained with DAPI.

Supplementary Figures of Original Blots

Title: The histone deacetylase SIRT6 blocks myostatin expression and development of muscle atrophy.

Authors: Sadhana A. Samant, Abhinav Kanwal, Vinodkumar Pillai, Riyue Bao and Mahesh P. Gupta

Figure 1A

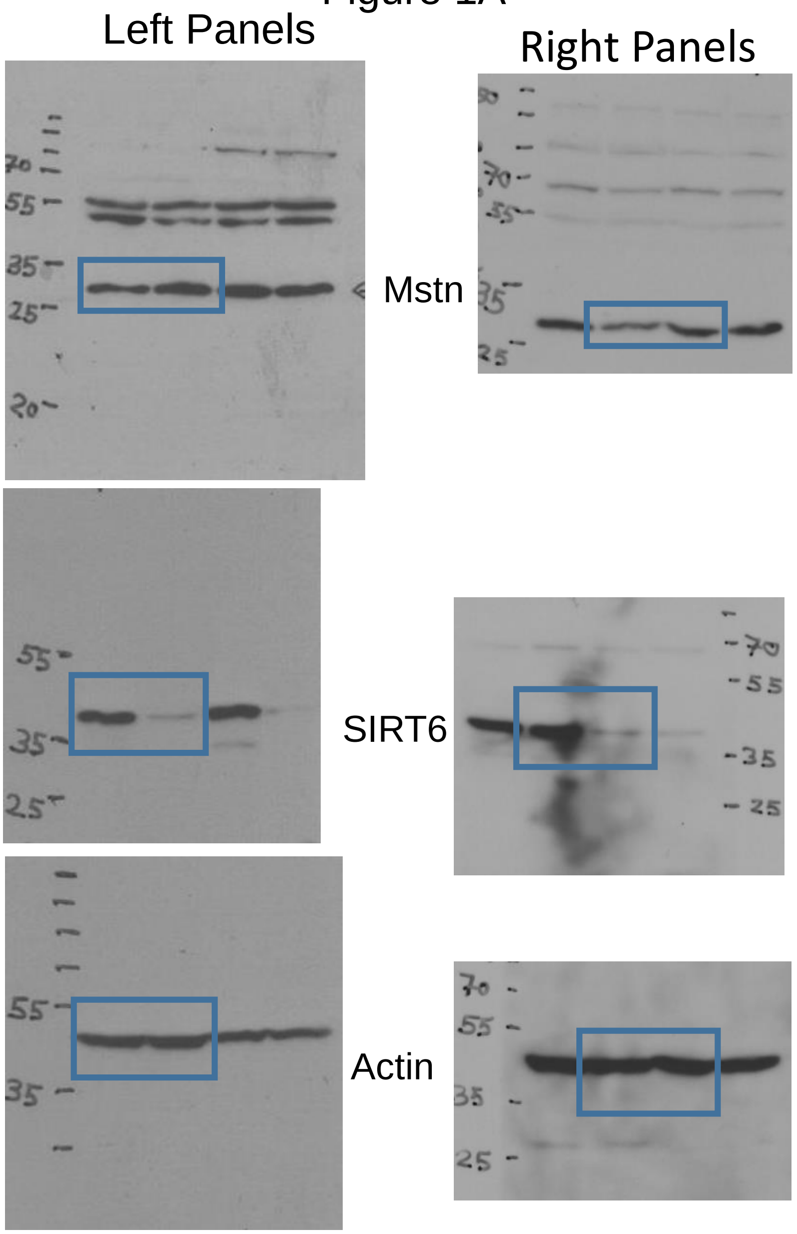

Figure 1G

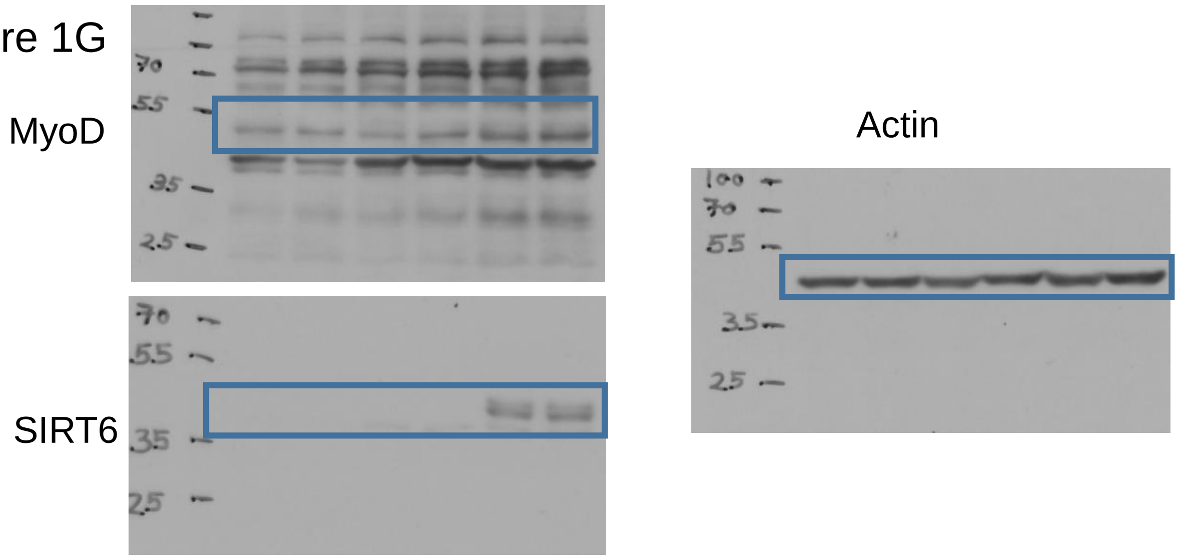

Figure 2B

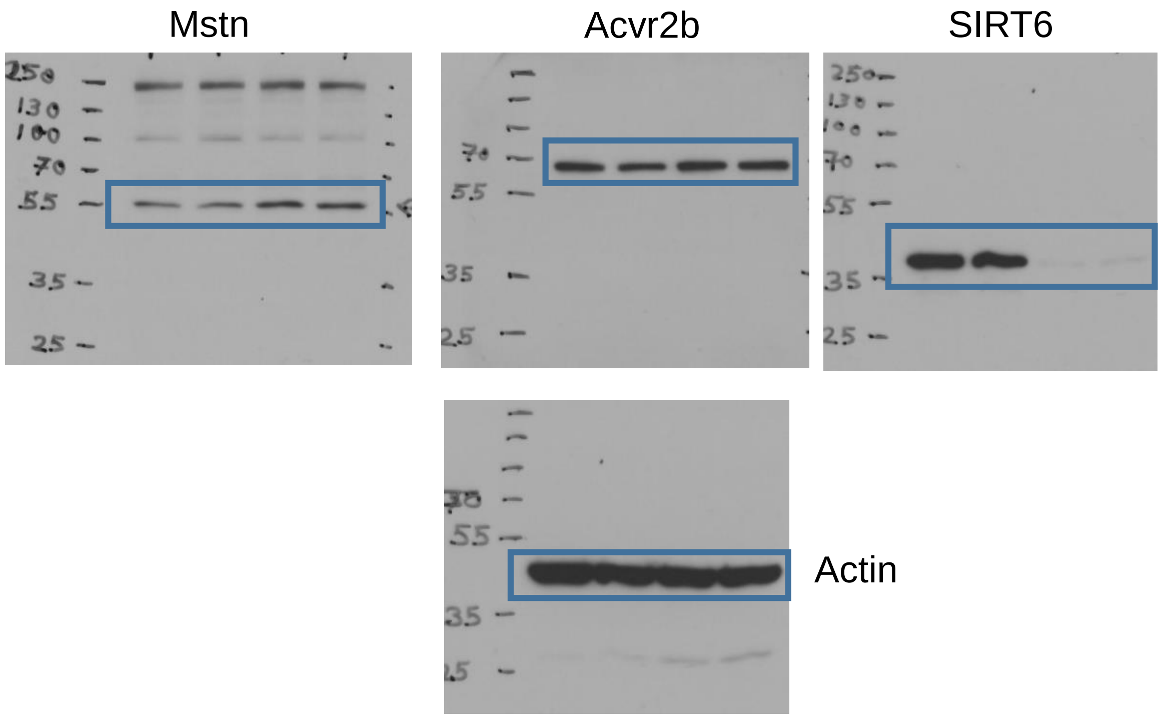

Figure 2F

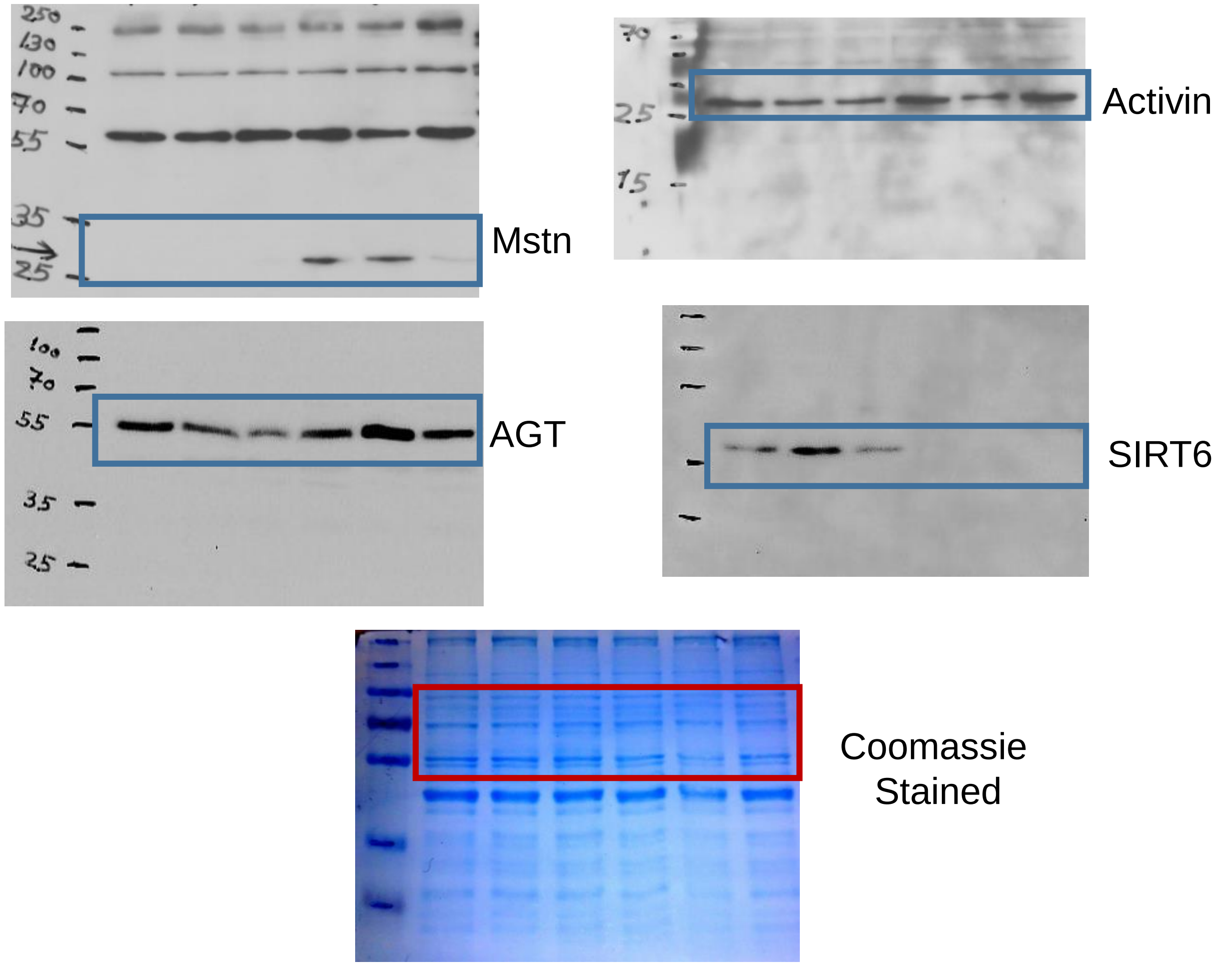

Figure 3B

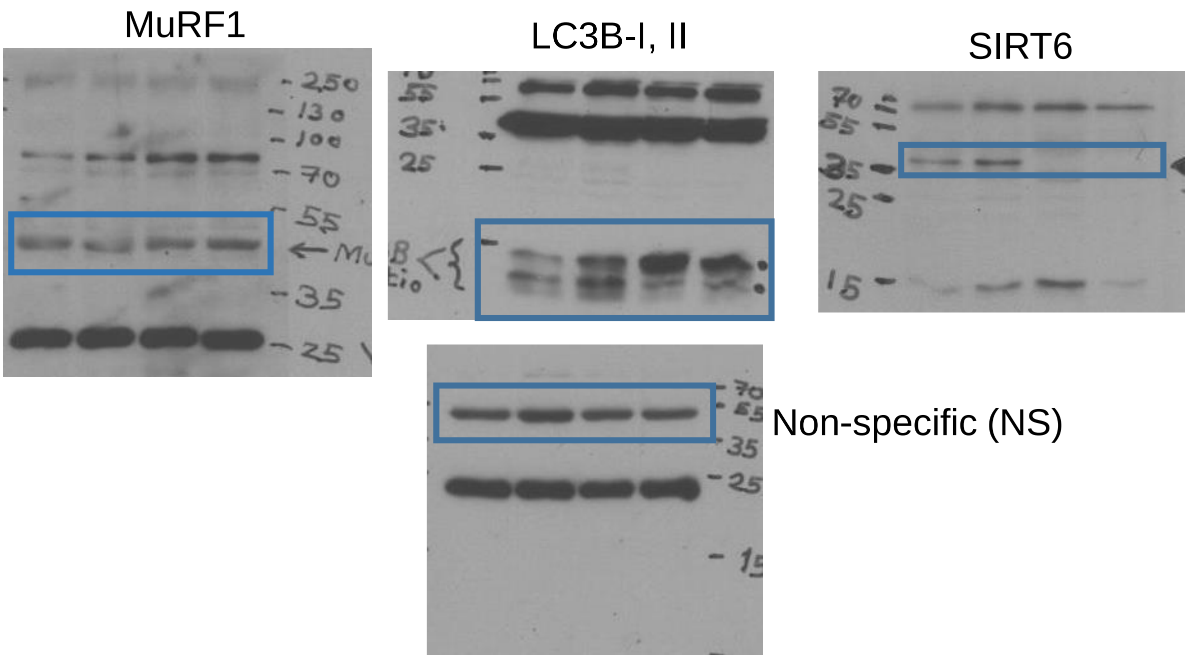

Figure 4B

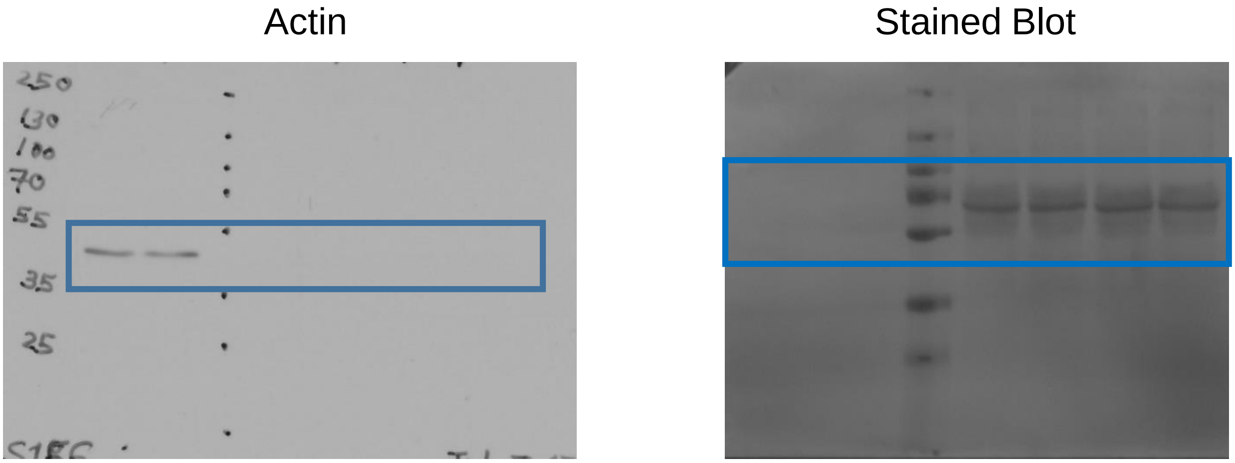

Figure 5A

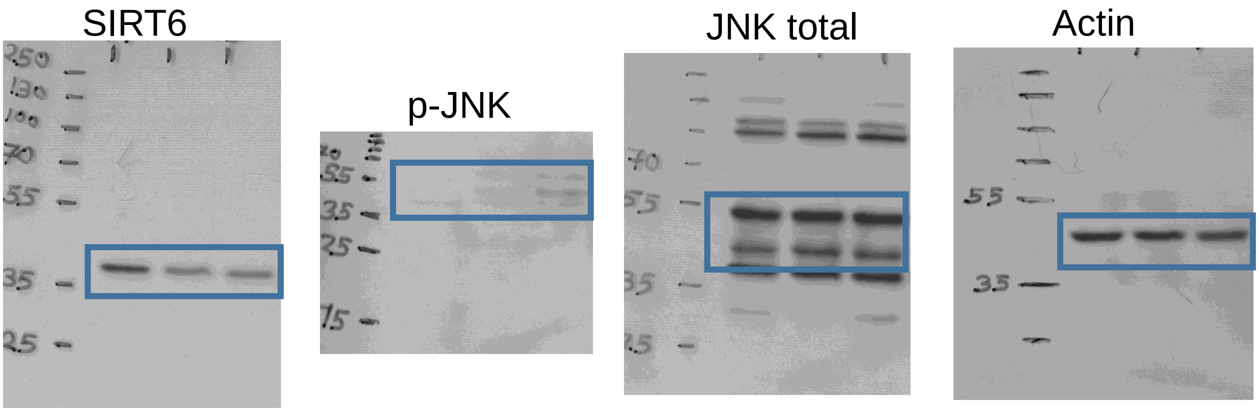

Figure 5B

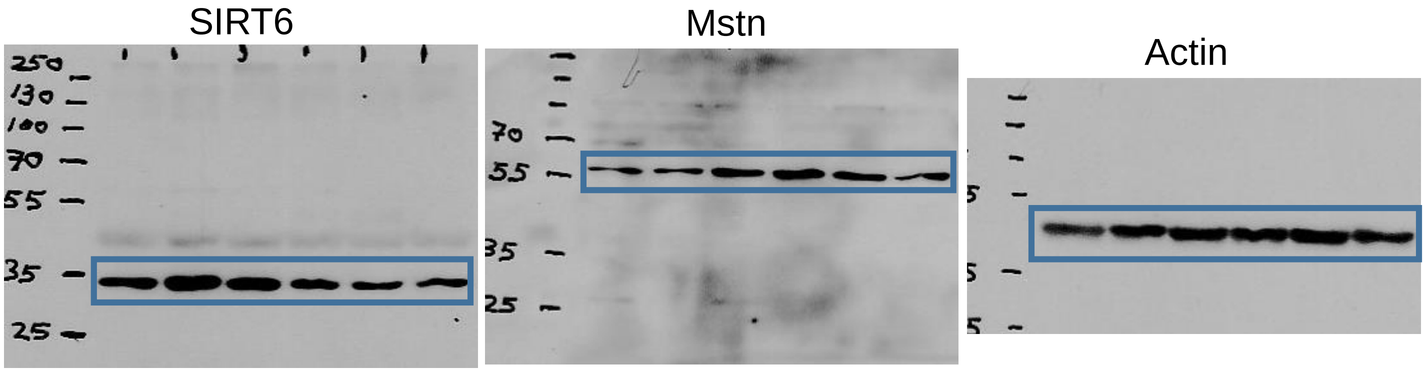

Figure 6B

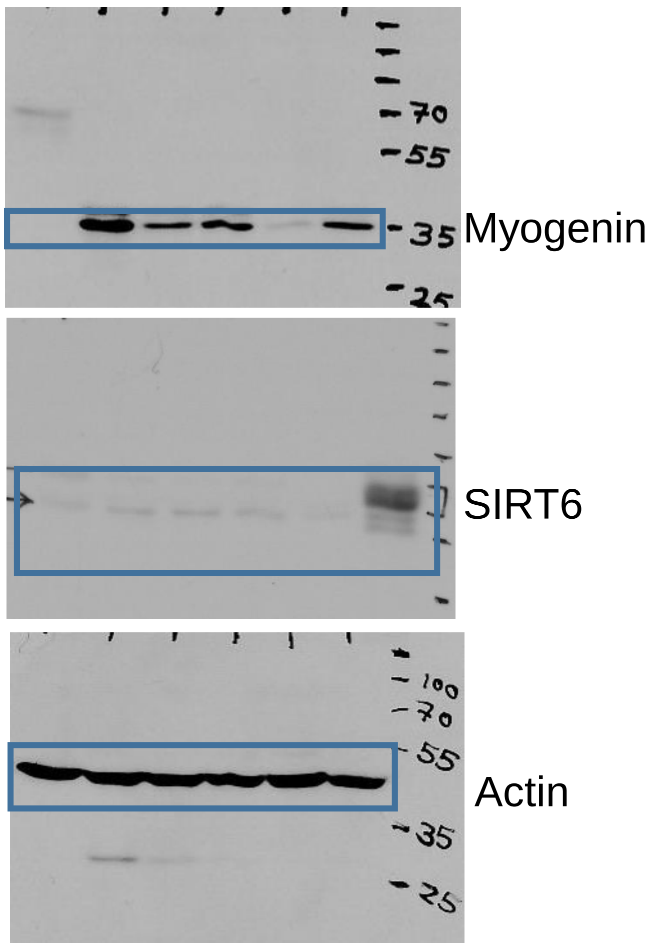

Figure 6C

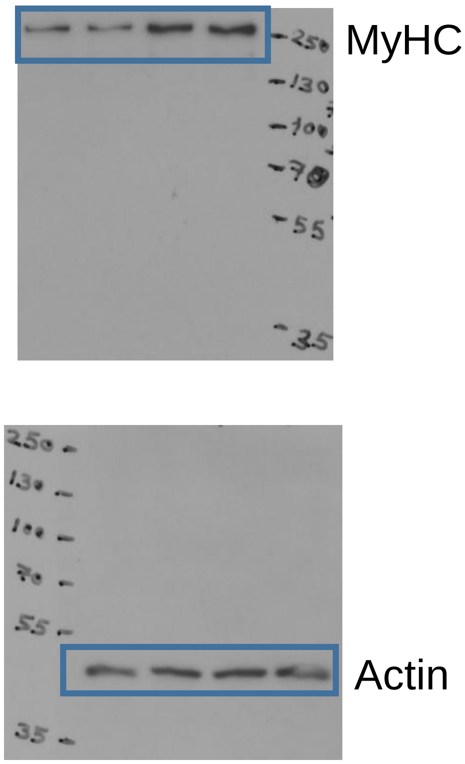

Figure 6C

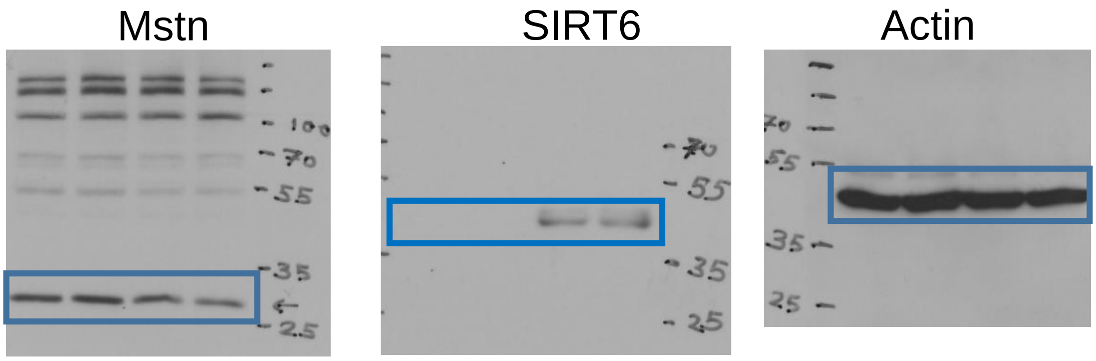

Supplement: Supplementary file 1 — Supplementary Information [file 41598_2017_10838_MOESM1_ESM.pdf]
